# A genomic perspective on the potential of *Actinobacillus succinogenes *for industrial succinate production

**DOI:** 10.1186/1471-2164-11-680

**Published:** 2010-11-30

**Authors:** James B McKinlay, Maris Laivenieks, Bryan D Schindler, Anastasia A McKinlay, Shivakumara Siddaramappa, Jean F Challacombe, Stephen R Lowry, Alicia Clum, Alla L Lapidus, Kirk B Burkhart, Victoria Harkins, Claire Vieille

**Affiliations:** 1 Department of Microbiology and Molecular Genetics, 2215 Biomedical Biophysical Sciences building, Michigan State University, East Lansing, MI 48824, USA; 2 Department of Genome Sciences & Medicine, University of Washington, Seattle, WA 98195, USA; 3 DOE Joint Genome Institute and Los Alamos National Laboratory, Los Alamos, NM 87545, USA; 4 DOE Joint Genome Institute, Walnut Creek, CA 94598, USA; 5 Department of Biochemistry and Molecular Biology, Michigan State University, East Lansing, MI 48824, USA; 6 Department of Microbiology, University of Washington in Seattle, WA 98195, USA; 7 Laboratory of Genetics, University of Wisconsin, Madison, WI 53706, USA; 8 Department of Zoology, Michigan State University, East Lansing, MI 48824, USA

## Abstract

**Background:**

Succinate is produced petrochemically from maleic anhydride to satisfy a small specialty chemical market. If succinate could be produced fermentatively at a price competitive with that of maleic anhydride, though, it could replace maleic anhydride as the precursor of many bulk chemicals, transforming a multi-billion dollar petrochemical market into one based on renewable resources. *Actinobacillus succinogenes *naturally converts sugars and CO_2 _into high concentrations of succinic acid as part of a mixed-acid fermentation. Efforts are ongoing to maximize carbon flux to succinate to achieve an industrial process.

**Results:**

Described here is the 2.3 Mb *A. succinogenes *genome sequence with emphasis on *A. succinogenes*'s potential for genetic engineering, its metabolic attributes and capabilities, and its lack of pathogenicity. The genome sequence contains 1,690 DNA uptake signal sequence repeats and a nearly complete set of natural competence proteins, suggesting that *A. succinogenes *is capable of natural transformation. *A. succinogenes *lacks a complete tricarboxylic acid cycle as well as a glyoxylate pathway, and it appears to be able to transport and degrade about twenty different carbohydrates. The genomes of *A. succinogenes *and its closest known relative, *Mannheimia succiniciproducens*, were compared for the presence of known Pasteurellaceae virulence factors. Both species appear to lack the virulence traits of toxin production, sialic acid and choline incorporation into lipopolysaccharide, and utilization of hemoglobin and transferrin as iron sources. Perspectives are also given on the conservation of *A. succinogenes *genomic features in other sequenced Pasteurellaceae.

**Conclusions:**

Both *A. succinogenes *and *M. succiniciproducens *genome sequences lack many of the virulence genes used by their pathogenic Pasteurellaceae relatives. The lack of pathogenicity of these two succinogens is an exciting prospect, because comparisons with pathogenic Pasteurellaceae could lead to a better understanding of Pasteurellaceae virulence. The fact that the *A. succinogenes *genome encodes uptake and degradation pathways for a variety of carbohydrates reflects the variety of carbohydrate substrates available in the rumen, *A. succinogenes*'s natural habitat. It also suggests that many different carbon sources can be used as feedstock for succinate production by *A. succinogenes*.

## Background

*Actinobacillus succinogenes *is a Gram-negative capnophilic bacterium that was isolated from bovine rumen as part of a search for succinate-producing bacteria [1]. Succinate is an important metabolic intermediate in the rumen, where several bacteria obtain energy by decarboxylating succinate to propionate, which in turn serves as a nutrient for the ruminant [2,3]. Succinate is used as a specialty chemical in food, agriculture, and pharmaceutical industries, but it has a much greater potential value for augmenting or replacing a multi-billion dollar petrochemical-based bulk chemical market [4,5]. Succinate production by fermentation of renewable feedstocks is both economically and environmentally attractive. A further environmental benefit is that fermentative succinate production uses CO_2_, a greenhouse gas, as a substrate.

*A. succinogenes *is one of the best succinate producers ever described, but it also produces formate and acetate in high concentrations. Flux distribution between succinate and alternative fermentation products is affected by environmental conditions. For example, higher succinate yields can be obtained by increasing the available CO_2 _and a reductant (e.g., by supplying H_2 _or by using carbon sources that are more reduced than glucose) [6]. Optimizing the environmental conditions is not sufficient to achieve a homosuccinate fermentation, though. Engineering *A. succinogenes*'s metabolism for homosuccinate production will be most effective if based on an understanding of the enzymes and mechanisms controlling flux distribution. Deciphering the *A. succinogenes *genome sequence is thus invaluable for defining, understanding, and engineering *A. succinogenes *metabolic pathways.

There is also much knowledge to be gained by comparing the *A. succinogenes *genome to its closest relatives. *A. succinogenes *is a member of the Pasteurellaceae family, which contains thirteen named genera as well as candidates for new taxa [7]. The best known genera are *Actinobacillus, Haemophilus*, and *Pasteurella*. At least thirty-two Pasteurellaceae genome sequences (complete and draft) are publicly available, fifteen of which are from different *H. influenzae *strains. While most Pasteurellaceae are studied for their pathogenic traits, *A. succinogenes *and its closest relative, *Mannheimia succiniciproducens *[8], collectively referred to as "succinogens" in this paper, are studied for their industrially attractive metabolic trait of succinate production. It will be important to confirm lack of pathogenicity in these succinogens before they are recommended for use on an industrial scale. Because *A. succinogenes *and *M. succiniciproducens *have never been reported in association with any disease, searching their genome sequences for Pasteurellaceae pathogenicity genes is a logical starting point to assess their potential for non-pathogenicity.

Here we present the first detailed analysis of the *A. succinogenes *genome sequence with a biotechnological perspective. The *A. succinogenes *and *M. succiniciproducens *genome sequences are also examined for known Pasteurellaceae virulence genes.

## Methods

### Chemicals, source strain, growth conditions, and genomic DNA purification

All chemicals were purchased from Sigma-Aldrich (St. Louis, MO). *A. succinogenes *type strain 130Z (ATCC 55618) was obtained from the American Type Culture Collection (Manassas, VA). To identify the *A. succinogenes *vitamin auxotrophies, *A. succinogenes *was grown in the defined medium, AM3 [9], and then transferred (1:100 dilutions) in parallel into ten tubes containing fresh AM3 medium, each tube lacking a single vitamin. *A. succinogenes *was considered prototrophic for a vitamin, if growth was maintained for three consecutive transfers in its absence. To confirm the minimal vitamin requirements, *A. succinogenes *was grown through seven transfers in AM3 containing only the required vitamins. To determine *A. succinogenes*'s ability to grow on various carbon sources, cells were grown anaerobically in Medium B (g/L: NaH_2_PO_4_·H_2_O, 8.5; K_2_HPO_4_, 15.5; bactotryptone, 10.0; yeast extract, 5.0; and NaHCO_3_, 2.1) supplemented with a single carbon source (1 g/L). The initial pH was adjusted to 7.0-7.2. *A. succinogenes *was considered able to grow on a carbon source when cell yields (absorbance at 660 nm) were higher in medium B supplemented with that carbon source than in non-supplemented medium. Growth data were recorded after each of two serial transfers of three biological replicates. For genomic DNA extraction, *A. succinogenes *was grown in 100 mL of tryptic soy glucose broth (Becton Dickinson, Sparks, MD) with 25 mM NaHCO_3 _in a 160-mL anaerobic serum vial at 37°C. The culture was harvested in log phase (~7.7 × 10^10 ^cells) and washed twice in 45 mL of phosphate buffer (g/L: K_2_HPO_4_, 15.5; NaH_2_PO_4_*H_2_O, 8.5; NaCl, 1). Genomic DNA was purified using a Qiagen genomic tip protocol with a Qiagen maxiprep column (Valencia, CA) as described in the QIAGEN Genomic DNA Handbook.

### Genome sequencing and assembly

Sequencing was performed by the Department of Energy's Joint Genome Institute (JGI). The genome of *A. succinogenes *was sequenced using a combination of three Sanger genomic libraries: 3 kb pUC18c, 8 kb pMCL200, and 40 kb fosmid libraries. All general aspects of library construction and sequencing performed at the JGI can be found at the JGI website [10]. 41,370 Sanger reads were assembled using PGA assembler (Paracel Genome Assembler 2.6.2, Paracel, Pasadena, CA). Possible mis-assemblies were corrected and gaps between contigs were closed by custom primer walks from sub-clones or PCR products. A total of 2,986 additional reactions were necessary to close gaps and to raise the quality of the finished sequence. The completed genome of *A. succinogenes *130Z contains 43,200 reads. The error rate of the finished genome sequence is less than 1 in 100,000. Together all libraries provided 11× coverage of the genome. The genome sequence of *A. succinogenes *strain 130Z is available in GenBank under accession number CP000746.

### Automated annotation

Automated annotation was performed by the Oak Ridge National Laboratory [11]. Open reading frames (ORFs) were identified using three gene caller programs: Critica, Generation, and Glimmer. Translated ORFs were subjected to an automated basic local alignment search tool (BLAST) for proteins [12] against GenBank's non-redundant database. The translated ORFs were also subjected to searches against KEGG, InterPro (incorporating Pfam, PROSITE, PRINTS, ProDom, SmartHMM, and TIGRFam), and Clusters of Orthologous Groups of proteins (COGs).

### Manual annotation

The ORFs described in this paper have also been manually annotated. BLAST alignments were examined to assess the correctness of the start codon. DNA sequences upstream of each ORF were examined for a ribosomal binding site (at least 3 nt of the AAGGAGG sequence, 5-10 nt upstream of the start codon) using the web Artemis tool [11]. To assign product names to each ORF, results from BLAST, HMM (i.e., PFAM and TIGRFAM), and domain and motif searches were considered. Most importantly, efforts were made to find a citation of biological function for a homologous gene. If a translated ORF was at least 75% identical to a protein of known function over 75% of the length, or if it belonged to a TIGRFAM equivalog, it was given the associated product name. If a translated ORF was less than 75% identical to a protein of known function, the product name was modified as follows: 60-75% identity over 65% of the length, putative product; 40-64% identity over 40% of the length, probable product; 25-39% identity over 25% of the length, possible product. If a translated ORF was at least 60% identical to a protein of unknown function, it was named a conserved hypothetical protein. If there was no adequate alignment with any protein (less than 25% identity or aligned region is less than 25% of the product length), the translated ORF was named a hypothetical protein.

### Other genome analyses

To compare their gene contents, the *A. succinogenes *and *M. succiniciproducens *genomes were re-annotated using the fully automated, prokaryotic genome annotation service, RAST (Rapid Annotation using Subsystem Technology) [13]. Pairwise BLAST comparisons of protein sets encoded by *A. succinogenes *and *M. succiniciproducens *genomes and predictions of the number of subsystems were performed using the sequence-based comparison tool available in RAST. Orthologous protein-coding genes in the two succinogens were manually compiled by comparing gene order, gene orientation (forward/reverse), features of intergenic regions, and protein similarity (minimum 25% identity at the protein level).

NUCmer and PROmer [14] whole-genome alignments were performed using an online Synteny plot tool [15]. Clustered regularly interspaced short palindromic repeats (CRISPR) and spacers were identified using the CRISPRs web service [16-18]. Spacer sequences were then aligned against the *A. succinogenes *genome sequence using BLAST. 16 S rRNA phylogeny was determined using the Michigan State University Ribosomal Database Project tools [19,20]. Hierarchical clustering of Pasteurellaceae genomes was done using tools at the JGI's Integrated Microbial Genomes (IMG) website [21,22].

Uptake signal sequence (USS) 9-mer cores [23] were counted, and their surrounding sequences were reported using our perl script, 200804USS.pl. The output was pasted into a Microsoft Excel spreadsheet to calculate the frequency of each nucleotide occurring at each position, upstream and downstream of the USS core. A search of *A. succinogenes *and *M. succiniciproducens *genomes for Pasteurellaceae virulence genes was performed by compiling a list of known Pasteurellaceae virulence genes based on the literature, then using a custom Python script to align their sequences against the two genomes using BLAST and report the data for the top hit.

## Results and Discussion

### General features

Even though it is one of the largest Pasteurellaceae genomes sequenced to date (Table 1), *A. succinogenes*'s genome is relatively small (2,319,663 bp, GenBank accession number CP000746). A total of 2,199 genes have been annotated in the genome, of which only 2,079 are protein-coding, a desirable feature for metabolic engineering. General features of *A. succinogenes*'s genome are compared to those of fifteen other Pasteurellaceae genomes in Table 1.

**Table 1:** General features of the *A. succinogenes *genome compared to fifteen^a ^complete Pasteurellaceae genomes

	*A. succinogenes*	Pasteurellaceae average^b^	Pasteurellaceae range^b^
Chromosome size (bp)	2,319,663	2,115,821	1,698,955-2,331,981
Genes	2,199	2,049	1,695-2,448
Protein-coding genes	2,079	1,940	1,619-2,369
Gene density (genes/kb)	0.945	0.969	0.912-1.082
Coding (%)	87	85	75-89
GC content (%)	44.9	39.8	37.2-44.9
5 S rRNAs	7	7	6-8
16 S rRNAs	6	5	3-6
23 S rRNAs	6	6	5-7

^a ^*A. aphrophilus *NJ8700; *A. pleuropneumoniae *strains AP76, JL03, and L20; *A. succinogenes *130Z; *H. ducreyi *35000HP; *H. influenzae *strains PittEE, PittGG, Rd KW20, and 86-028NP; *H. parasuis *SH0165; *H. somni *strains129PT and 2336; *M. succiniciproducens *MBEL55E; and *P. multocida *Pm70.

^b ^Data based on the National Center for Biotechnology Information (NCBI) genome summary pages.

The *A. succinogenes *genome is most closely related to that of its succinogen relative, *M. succiniciproducens *[8] (additional file 1: Figures S1 and S2). *M. succiniciproducens *was also isolated from a bovine rumen, albeit on a different continent, and it shares many metabolic traits with *A. succinogenes *(see below). Based on genome re-annotations performed with RAST (2,223 protein-encoding genes, total), the two succinogens' genomes have 1,735 ORFs (78%) in common, 442 ORFs are found only in *M. succiniciproducens*, and 488 ORFs are found only in *A. succinogenes*. Of 2,081 automated KEGG comparisons [24], 1,861 (89%) *A. succinogenes *genes were most similar to other Pasteurellaceae genes, with 1,252 (60%) being most similar to *M. succiniciproducens *genes. However, *A. succinogenes *and *M. succiniciproducens *are among at least twenty-four misclassified Pasteurellaceae species that will likely be renamed, as they do not cluster with properly classified species in phylogenetic trees based on Pasteurellaceae 16 S rRNA, *infB*, *rpoB, or atpD *gene sequences [25]. The two succinogens are often clustered together using phylogenetic approaches, but not closely enough to suggest that they belong to the same genus [25]. Hierarchical clustering of gene function categories also places the two succinogens in a clade separate from other Pasteurellaceae (additional file 1: Figure S2). To better gauge how closely related the two organisms are, we performed whole genome NUCmer and PROmer alignments of the two succinogens with each other, as well as with eight other Pasteurellaceae (additional file 1: Figure S3). NUCmer plots show little to no conservation of genome structure at the nucleotide level between *A. succinogenes *and any other *Pasteurellaceae*. PROmer plots reveal that *A. succinogenes *and *M. succiniciproducens *are more related to each other than to other Pasteurellaceae. Overall, though, the PROmer plots show that drastic changes in genome structure have occurred as *A. succinogenes *and *M. succiniciproducens *evolved divergently from their last common ancestor, and that the two succinogens are more distantly related than their functional traits would suggest.

#### Prophage

A 39,489-bp prophage genome is encoded in the Asuc_1205-58 region. The presence of a prophage has biotechnological relevance for two reasons. First, it raises the possibility of using phage-based genetic engineering. Second, it suggests that *A. succinogenes *may be susceptible to phage lysis in an industrial bioreactor; if so, steps should be taken to eliminate this prophage from the host genome. This prophage has an organization similar to that of the *Aggregatibacter actinomycetemcomitans *phage, AaΦ23 [26], and it contains a DNA N-6-adenine-methyltransferase (Asuc_1221). The *A. succinogenes *prophage differs from AaΦ23, though, in that the integrase gene (Asuc_1258) is located at the opposite end of the phage genome from its location in AaΦ23. Despite sharing a similar organization, many of the *A. succinogenes *phage proteins are not found in AaΦ23, and they are conserved in only a few Pasteurellaceae genomes. For example, the Asuc_1233-44 proteins are not well conserved among Pasteurellaceae, but they include such crucial proteins as both terminase subunits (Asuc_1235-6), a portal protein (Asuc_1238), a prohead protease (Asuc_1239), a major capsid protein (Asuc_1240), a protein with possible DNA-packing function (Asuc_1241), and a putative head-tail adaptor (Asuc_1242). Two sets of addiction module killer and antidote proteins are also encoded (Asuc_1211-4). Another interesting feature of the *A. succinogenes *phage is that Asuc_1219, encoding a homolog to replication protein O (which, along with the P protein [Asuc_1220], initiates lytic replication), has an internal frame shift. It is unclear at this point whether this prophage corresponds to a functional temperate phage. Growth experiments in the presence of mitomycin C showed that 0.1 μg/mL mitomycin C started inhibiting growth after 3 h, 1 μg/mL mitomycin C inhibited growth starting at 90 min, and no growth was observed at 10 μg/mL mitomycin C. These results are similar to those observed for phage induction in *E. coli *K12 [27]. More work (beyond the scope of this paper) would be needed, though, to demonstrate that the inhibitory effect of mitomycin C is associated with the release of phage particles in the culture broth.

A set of CRISPRs (located between Asuc_1293 and 1294) and a set of CRISPR-associated genes (Asuc_1284-93) are located thirty-four ORFs downstream from the prophage. Together, these CRISPRs and CRISPR-associated genes can provide resistance against phage [28]. Homologs in the 30% to 75% identity range to *A. succinogenes *CRISPR-associated genes are found in *M. succiniciproducens *and *Mannheimia haemolytica*, but they are not found in other Pasteurellaceae. Short fragments of five of the ten *A. succinogenes *CRISPR spacers matched short sequences in *A. succinogenes *prophage genes. One spacer sequence showed similarity to a gene encoding a phage integrase family protein (Asuc_0030). This gene is accompanied by only one other phage gene encoding a probable capsid portal protein Q with two internal frame shifts (Asuc_0029, 58% identical to an *M. haemolytica *phage ΦMHaA1 protein and 73% identical to a phage protein found in several *H. influenzae *genomes). These two genes may be the last remnants of an ancient phage integration-excision event.

*A. succinogenes*, *M. succiniciproducens*, and nine other Pasteurellaceae species contain a homolog of *Escherichia coli *LamB (Asuc_0322, 54% identical), the maltoporin functioning as receptor for phage lambda. In both succinogens, the *lamB *homolog is part of the maltose transport operon (additional file 2: Table S1). This gene is likely functional in *A. succinogenes*, since *A. succinogenes *grows well on maltose [1]. This result suggests that *A. succinogenes *may be susceptible to infection by lambda-related bacteriophages.

### Natural competence

All Pasteurellaceae genomes contain USS repeats that feature a conserved 9-nt sequence [29]. In most Pasteurellaceae the conserved 9-mer is AAGTGCGGT (i.e., USS1), whereas ACAAGCGGT (i.e., USS2) predominates in *Actinobacillus pleuropneumoniae, H. ducreyi, H. parasuis *and *M. haemolytica *[29]. Each USS is followed by a conserved AT-rich region, and USS2 is additionally followed by GCAAA(A/T) 20-nt downstream of the 9-mer [29] (additional file 1: Figure S4). The only function yet demonstrated for USS repeats is in natural competence [30]. Under certain conditions (e.g., starvation or the presence of elevated cAMP levels) many Pasteurellaceae preferentially internalize USS-containing DNA, perhaps being recognized as self-DNA [29,31]. Uptake of USS-containing DNA is facilitated by a number of competency proteins, resulting in transformation frequencies that can be as high as 10^-3 ^to 10^-2 ^transformants per CFU [31] and in homologous recombination with chromosomal DNA. This DNA uptake mechanism works best with linear DNA, making it well suited for strain engineering, since constructs can be easily generated by PCR and double recombination events are needed to integrate the linear DNA into the chromosome. Genetic tools for *A. succinogenes *are currently limited to expression vectors [32,33]. The ability to replace chromosomal DNA with engineered DNA is invaluable for making gene knockouts (e.g., to block unwanted fermentation pathways) and knock-ins (e.g., of a modified promoter to affect enzyme expression).

**Figure 1:**
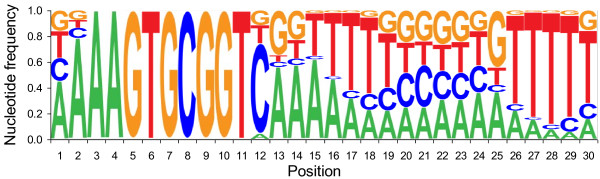
**Nucleotide frequency in *A. succinogenes *USS repeats**.

We examined the *A. succinogenes *genome sequence and all other complete Pasteurellaceae genome sequences for USS occurrences (as of September 2009). All Pasteurellaceae favored either USS1 or USS2, but not both. In all cases, USS sequences were roughly equally distributed between each DNA strand. *A. succinogenes *has a USS density of 0.73 USS/kb and contains 1,690 USS1 repeats; only *Aggregatibacter aphrophilus *and *A. actinomycetemcomitans *had more (Table 2). As in other Pasteurellaceae (additional file 1: Figure S4), the *A. succinogenes *9-mer core is usually preceded by an A and followed by an AT-rich region (Figure 1). One outstanding difference is that the *A. succinogenes *9-mer core is immediately followed by a C in 71% of the USS repeats. The frequency of C in this position ranges from 27% in *Histophilus somni *to 51% in *A. aphrophilus *for USS1, and from 36% in *H. ducreyi *to 57% in *A. pleuropneumoniae *for USS2 (Table 2).

**Table 2:** Occurrence of USS repeats in complete Pasteurellaceae genome sequences

Strain name^a^	Genome size (nt)	USS1: AAGTGCGGT	USS2: ACAAGCGGT	C at nt 12 (%)^b^	Density (USS/kb)
*A. actinomycetemcomitans*	2,105,332	1760	43	44	0.84
*H. influenzae *Rd KW20	1,830,138	1471	56	30	0.80
*A. aphrophilus *NJ8700	2,313,035	1857	37	51	0.80
*H. influenzae *PittEE	1,813,033	1450	55	29	0.80
*H. influenzae *PittGG	1,887,192	1498	53	30	0.79
*H. influenzae *86-028NP	1,914,490	1516	51	29	0.79
***A. succinogenes *130Z**	**2,319,663**	**1690**	**73**	**71**	**0.73**
*M. succiniciproducens*	2,314,078	1485	95	44	0.64
*H. somni *129PT	2,007,700	1244	48	27	0.62
*H. somni *2336	2,263,857	1355	47	27	0.60
*P. multocida *str. Pm70	2,257,487	927	41	46	0.41
*A. pleuropneumoniae *str. JL03	2,242,062	75	767	55	0.34
*A. pleuropneumoniae *L20	2,274,482	73	765	57	0.34
*A. pleuropneumoniae *str. AP76	2,331,981	74	781	55	0.33
*H. parasuis *SH0165	2,269,156	109	523	37	0.23
*H. ducreyi *35000HP	1,698,955	41	199	36	0.12

^a ^Arranged according to descending USS density. USS densities are listed for the most common USS in each genome.

^b ^For most common USS in genome only.

A regulon of twenty-five competency genes is known in *H. influenzae *[31]. The *A. succinogenes *genome contains homologs of all of these genes except for two, HI0660 and HI1631, which encode hypothetical proteins in *H. influenzae*. Seven of the *A. succinogenes *competency proteins are less than 40% identical to their *H. influenzae *homologs (additional file 2: Table S2). In addition to twenty-three competency genes, the *A. succinogenes *genome also encodes the master regulator of the competence regulon (the cAMP receptor protein, Asuc_0008) and the essential competence regulatory factor, Sxy (Asuc_0283). It also encodes proteins that are not competence-induced, but that are known to participate in DNA uptake or recombination (i.e., RecA, TopA, AtpA, and DsbA, additional file 2: Table S2) [30]. The abundance of USS repeats in *A. succinogenes *and the possible presence of the necessary machinery for natural competence suggested that *A. succinogenes *could be naturally competent. Recent experiments in our laboratory demonstrated that *A. succinogenes *could uptake DNA by natural transformation. These transformations led to the construction of two gene knockouts (Joshi et al., manuscript in preparation).

### Metabolic reconstruction

#### Central metabolism

**Figure 2:**
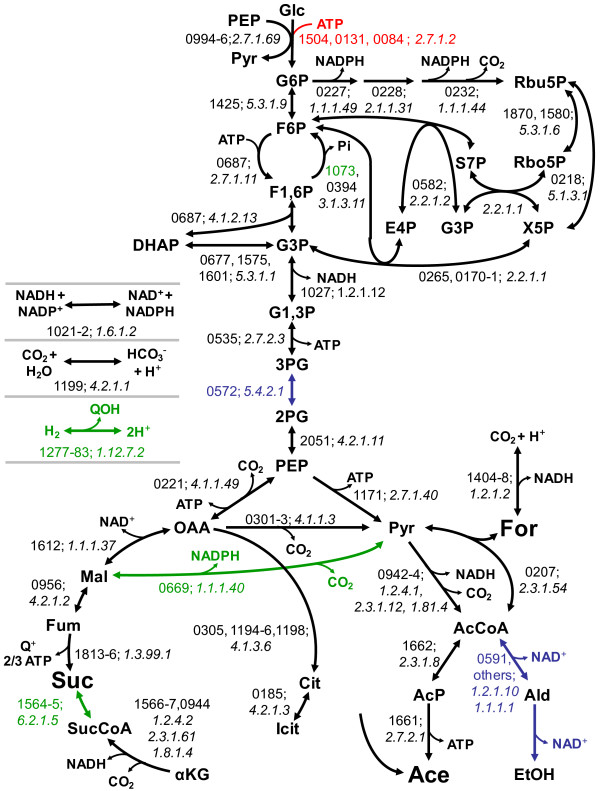
***A. succinogenes *central metabolic network based on annotation of metabolic genes**. Four-digit numbers are Asuc_ORF (locus tags) numbers and are followed by E.C. numbers. Hyphenated locus tag numbers indicate that the enzyme is encoded by several successive genes. Reaction names: see additional file 2: Table S3. Arrow and number colors: black, product function assumed; green, putative function assumed; blue, probable function assumed; red, possible function assumed. Metabolites: AcCoA, acetyl-CoA; Ace, acetate; AcP, acetyl-phosphate; Ald, acetaldehyde; Cit, citrate; EtOH, ethanol; E4P, erythrose-4-phosphate; For, formate; Fum, fumarate; F1,6P, fructose-1,6-bisphosphate; F6P, fructose-6-phosphate; Glc, glucose; G1,3P, glycerate-1,3-bisphosphate; G3P, glyceraldehyde-3-phosphate; G6P, glucose-6-phosphate; Icit, Isocitrate; αKG, α-ketoglutarate; Mal, malate; OAA, oxaloacetate; PEP, phosphoenolpyruvate; 2PG, 2-phosphoglycerate; 3PG, 3-phosphoglycerate; Pyr, pyruvate; Q^+^, menaquinone; QOH, menaquinol; R5P, ribose-5-phosphate, Ru5P, ribulose-5-phosphate, Suc, succinate; SucCoA, succinyl-CoA; S7P, sedoheptulose-7-phosphate; X5P, xylulose-5-phosphate.

A complete inventory of *A. succinogenes*'s metabolic machinery is crucial for understanding and engineering the pathways that are involved in succinate production. *A. succinogenes*'s metabolism has been studied using fermentation balances, enzyme assays, and ^13^C-metabolic flux analyses, primarily in glucose-grown cultures [6,9,34,35]. These studies indicated that glucose uptake takes place both through a permease (followed by glucose phosphorylation by hexokinase) and through the phosphoenolpyruvate (PEP)-dependent phosphotransferase system (PTS). Glucose-6-phosphate is then catabolized to phosphoenolpyruvate (PEP) via glycolysis, with little involvement of the pentose phosphate pathway. PEP is then converted into fermentation products via the C_3 _pathway (leading to formate, acetate, and ethanol) and the C_4 _pathway (leading to succinate), with malic enzyme and oxaloacetate (OAA) decarboxylase forming reversible shunts between these pathways. These studies also showed the absence of glyoxylate and Entner-Doudoroff pathway fluxes. The enzymes of central metabolism encoded in the genome are summarized in Figure 2 and additional file 2: Table S3. While the *A. succinogenes *genome encodes the EI, Hpr, and EIIA components of the PTS (Asuc_0994-96), it does not encode a homolog of *E. coli *EIIBC (PtsG). PTS-dependent glucose uptake in *A. succinogenes *might take place, instead, using the mannose-specific PTS proteins ManXYZ (Asuc_936-38). The PTS-independent glucose uptake mechanism is believed to be a major factor explaining *A. succinogenes*'s ability to produce large amounts of succinate, but the genes involved are not characterized at this point. The genome encodes a sugar transport protein (Asuc_0496) that shows 40% similarity to the *Zymomonas mobilis *glucose facilitator protein, as well as possible sugar kinases (Asuc_1504, 0131, and 0084). In agreement with previous studies [6,34], genes encoding all of the glycolytic and pentose phosphate pathway enzymes are present, whereas those encoding glyoxylate pathway enzymes are absent. While the gene encoding the Entner-Doudoroff enzyme phosphogluconate dehydratase is not present in the genome, three possible genes encoding 2-keto-3-deoxy-6-phosphogluconate (KDPG) aldolases were identified (Asuc_0152, 0374, and 1471). These three genes are part of operons encoding possible glucuronate or galacturonate degradation pathways. Because *A. succinogenes *did not grow on these two substrates in the conditions tested (see Materials and Methods), the functions of Asuc_0152, 0374, and 1471 remain unknown. These aldolases likely break down KDPG originating from yet unknown growth substrates rather than from the Entner-Doudoroff pathway. *M. succiniciproducens *was reported to have a complete Entner-Doudoroff pathway [8], which would be a significant difference between the two succinogens. However, the purported *M. succiniciproducens *phosphogluconate dehydratase (MS2219) is more likely the dihydroxy-acid dehydratase (IlvD) involved in branched-chain amino acid synthesis. In fact, BLASTP searches using the *E. coli *IlvD or phosphogluconate dehydratase (accession number NP_416365.1) as the query sequence identify the same top hit in most Pasteurellaceae proteomes. *E. coli *IlvD shares at least 75% identity with the top hit in each Pasteurellaceae species, while phosphogluconate dehydratase shares at most 30% identity with the same top hits. It is therefore unlikely that any Pasteurellaceae sequenced to date-- including *M. succiniciproducens*--has a full Entner-Doudoroff pathway.

All the genes encoding C_4 _pathway enzymes were identified in the *A. succinogenes *genome. In contrast to *M. succiniciproducens*, which contains both a PEP carboxykinase (PEPCK) and a PEP carboxylase [8], PEPCK is the only PEP-carboxylating enzyme in *A. succinogenes *(Asuc_0221). The other C_4 _pathway enzymes (i.e., malate dehydrogenase [Asuc_1612], fumarase [Asuc_0956], and fumarate reductase [Asuc_1813-6]) make up an incomplete arm of a reductive tricarboxylic acid cycle (TCA) that also includes succinyl-CoA synthetase (Asuc_1564-5). Unlike *M. succiniciproducens *[8], *A. succinogenes *lacks the TCA cycle enzymes citrate synthase and isocitrate dehydrogenase, but it does have an α-ketoglutarate (αKG) dehydrogenase (Asuc_1566-7 and 0944) and a citrate lyase (Asuc_0305, 1194-6 and 1198). Similar to the genome organization of *E. coli *[36], the *A. succinogenes *αKG dehydrogenase E3 subunit (i.e., lipoamide dehydrogenase) is also the pyruvate dehydrogenase E3 subunit, and it is encoded in the pyruvate dehydrogenase operon (Asuc_0942-4).

Most of the C_3 _pathway enzymes were identified, including pyruvate dehydrogenase and formate dehydrogenase (Asuc_1261-6) (Figure 2). Fluxes through these two dehydrogenases were shown to be important sources of reducing power for succinate production and for anabolism when coupled with transhydrogenase activity [34,35]. A single predicted membrane-bound transhydrogenase was also identified (Asuc_1021-22). The only uncertainty in the C_3 _pathway is the mechanism by which ethanol is produced from acetyl-CoA, but the genome contains a number of aldehyde and alcohol dehydrogenases. Among them, Asuc_0403, 0591, 1136, and 1955 belong to the iron-containing alcohol dehydrogenase protein family. Asuc_0591 is a good candidate for ethanol production. It is probably a multifunctional aldehyde/alcohol dehydrogenase [37], and it is 47% identical (66% similar) to *E. coli *AdhE (GenBank accession no. P0A9Q7), the enzyme responsible for ethanol production. Asuc_0067 encodes a class III alcohol dehydrogenase. A class III alcohol dehydrogenase functions primarily as a formaldehyde dehydrogenase (E.C. 1.1.1.284), but it can also produce ethanol [38]. Even though *M. succiniciproducens *is not known to produce ethanol, it has homologs of all the *A. succinogenes *alcohol dehydrogenases mentioned above. Thus, these proteins are either not functional for ethanol production in *M. succiniciproducens*, or they are not involved in ethanol production in *A. succinogenes*.

Metabolic flux distribution between the C_3 _and C_4 _pathways is known to be influenced by CO_2 _and H_2 _concentrations. Malic enzyme (Asuc_0669) and a sodium-pumping OAA decarboxylase (Asuc_0301-3), which are responsible for large reversible fluxes between the C_3 _and the C_4 _pathways [34], are encoded in the genome. Carbonic anhydrase (Asuc_1199), which interconverts CO_2 _and HCO_3_^-^, was identified, and it could be important for making CO_2 _available for succinate production in environments with different pH values. A single membrane-bound hydrogenase (Asuc_1277-83) was also identified.

In contrast to *M. succiniciproducens*, *A. succinogenes *does not produce lactate [6]. The *A. succinogenes *genome encodes a single lactate dehydrogenase, Asuc_0005, which is 60% identical to the *E. coli *enzyme (EC 1.1.1.28) that couples lactate oxidation to amino acid and sugar transport [39]. Asuc_0005 is therefore expected to oxidize, rather than generate, lactate. In contrast, the *M. succiniciproducens *genome does not encode an Asuc_0005 homolog. *A. succinogenes *was grown anaerobically in AM3 with 100 mM NaHCO_3-_, 50 mM glucose, and 25 mM D, L-lactate, but no lactate consumption was observed (data not shown).

*A. succinogenes *is also capable of gluconeogenesis, since it can grow by anaerobic respiration using H_2 _or electrically reduced neutral red as an electron donor and using fumarate or malate as the carbon source and electron acceptor [6,40,41]. The genome sequence encodes a putative type I (Asuc_1073) and a type II (Asuc_0394) fructose-1,6-bisphosphatase, but it does not encode a PEP synthase. As a result, *A. succinogenes *must rely on gluconeogenic flux through PEPCK to make PEP from malate and fumarate.

#### Auxotrophic features

Production of succinate in a chemically defined medium can decrease downstream costs in product purification. *A. succinogenes *is known to require glutamate, cysteine, and methionine to grow in a defined medium [9]. Glu auxotrophy is due to an inability to synthesize αKG from glucose [9], which is now explained by the absence of genes encoding isocitrate dehydrogenase in the genome sequence. αKG cannot be synthesized from succinate because of the unidirectional activity of αKG dehydrogenase from αKG to succinyl-CoA (ΔG°' = -30,000) and because the *A. succinogenes *genome does not encode the reductive-TCA cycle enzyme, αKG ferredoxin oxidoreductase. Surprisingly, *A. succinogenes *encodes all the enzymes required to synthesize Cys (additional file 2: Table S4). Since both Cys and Met are sulfur-containing amino acids, we wondered if these auxotrophies could be due to an inability to assimilate sulfate, the only mineral sulfur source in AM3. Indeed, the *A. succinogenes *genome does not encode adenylsulfate kinase (additional files 1 and 2: Figure S5 and Table S4), which is required for assimilatory sulfate reduction. *A. succinogenes *grew normally in AM3 once sodium sulfide or sodium thiosulfate was added in place of Cys, confirming that the Cys auxotrophy is due to an inability to reduce sulfate. Met, however, was still required for growth in the presence of reduced sulfur compounds. *A. succinogenes *is missing several genes necessary to synthesize Met through the L-homocysteine pathway, and it would require a source of methanethiol to produce Met from O-acetyl homoserine (additional files 1 and 2: Figure S5 and Table S4).

We determined that nicotinic acid, pantothenate, pyridoxine, and thiamine are the only four vitamins required by *A. succinogenes*. The *A. succinogenes *genome sequence is missing several genes involved in the biosynthesis of these vitamins (additional files 1 and 2: Figure S6 and Table S4). Even though it is missing several genes involved in biotin synthesis (e.g., *bioA*, *bioF*, and *bioW*), *A. succinogenes *grows repeatedly in the absence of biotin (five consecutive transfers in AM3). However, *A. succinogenes *grows more reliably in AM3 supplemented with biotin when inoculated from frozen stocks or from rich medium. Biotin can therefore be considered non-essential, but stimulatory, for the growth of *A. succinogenes*, a feature shared with *M. succiniciproducens *[42]. One mechanism that could explain the growth of *A. succinogenes *in the absence of biotin despite the absence of a full set of biotin biosynthetic genes is that *A. succinogenes *might be able to use thiamine as a precursor for biotin synthesis, as has been observed with the fungus, *Humicola*, strain 16-1 [43]. The *A. succinogenes *gene encoding a putative biotin synthase (Asuc_1132) seems to be co-transcribed with the thiamine ABC transporter genes (Asuc_1229-31).

Similar to *A. succinogenes, M. succiniciproducens *is auxotrophic for Cys, Met, nicotinic acid, pantothenate, pyridoxine, and thiamine, and the genetic bases underlying these auxotrophies are the same as those identified in *A. succinogenes *[42]. *A. succinogenes *and *M. succiniciproducens *differ in one respect, though. *M. succiniciproducens *is not auxotrophic for Glu since, unlike *A. succinogenes*, it has genes encoding a citrate synthase (MS2371) and an isocitrate dehydrogenase (MS2370). Although the two succinogens have an incomplete assimilatory sulfate reduction pathway, they, along with various *A. pleuropneumoniae *strains and *Actinobacillus minor *NM305, have the most complete assimilatory sulfate reduction pathway among all other sequenced Pasteurellaceae. The inability of *A. succinogenes *and *M. succiniciproducens *to carry out assimilatory sulfate reduction is likely an adaptation to their natural environment. The rumen flora produces hydrogen sulfide [44,45]. Both succinogens encode a serine acetyltransferase (Asuc_0384 and MS2212) and a cysteine synthetase (Asuc_2108 and MS1770) that may allow them to synthesize L-Cys from H_2_S produced in the rumen.

The insights into *A. succinogenes*'s auxotrophies obtained by genome analyses have allowed us to modify our original defined medium. Whereas Glu and Met are two of the least expensive amino acids (~$1/kg), cysteine is more expensive (> $10/kg) [46]. Using inexpensive inorganic reduced sulfur compounds (such as thiosulfate in place of cysteine) and eliminating several nonessential vitamins are expected to significantly reduce the cost of defined growth medium.

#### Dicarboxylic acid transporters

As a producer of the dicarboxylate succinate, it is interesting to note that *A. succinogenes *encodes twelve possible anaerobic dicarboxylate transporters (additional file 2: Table S5). Nine of them are similar to the tripartite ATP-independent periplasmic transporters (T.C. 2.A.56) encoded by *dctPQM *[47]. These transporters have been characterized in *Rhodobacter capsulatus *and *Wolinella succinogenes *for their roles during fumarate respiration, where fumarate is transported by proton symport [47,48]. The other three anaerobic dicarboxylate transporters are related to DcuA, B, and C (T.C. 2.A.13). These transporters have been characterized during fumarate respiration by *E. coli *and *W. succinogenes *[48-50]. They operate by exchanging an intracellular dicarboxylate (e.g., succinate) for an extracellular dicarboxylate (e.g., fumarate, malate, or aspartate). DcuA and B may also transport Na^+ ^in symport with the dicarboxylates to avoid dissipating the proton motive force [48]. DcuC may have preferential succinate efflux activity, since a *dcuC^,- ^E. coli *strain has increased dicarboxylate exchange and fumarate uptake activities [51]. During *E. coli *mixed acid fermentation, glucose did not repress *dcuC *expression, suggesting that DcuC plays a role in succinate excretion [51]. A microarray study examining changes in *H. influenzae *gene expression during competency induction suggested that DcuA and B are important for Pasteurellaceae fermentation or fumarate respiration [31]. In that study, 151 genes showed > 4-fold increase in transcript levels as *H. influenzae *became competent, including transcripts for the C_4 _pathway enzymes aspartate ammonia-lyase, malate dehydrogenase, fumarase, fumarate reductase, and a single dicarboxylate transporter. This *H. influenzae *transporter is 84% and 44% identical to *A. succinogenes*'s putative DcuB-type transporters, Asuc_0142 and 1999, respectively. *A. succinogenes dcuA*, *B*, and *C *are therefore candidate genes to investigate the importance of dicarboxylate transport during fumarate respiration and succinate fermentation.

#### Sugar transporters

**Figure 3:**
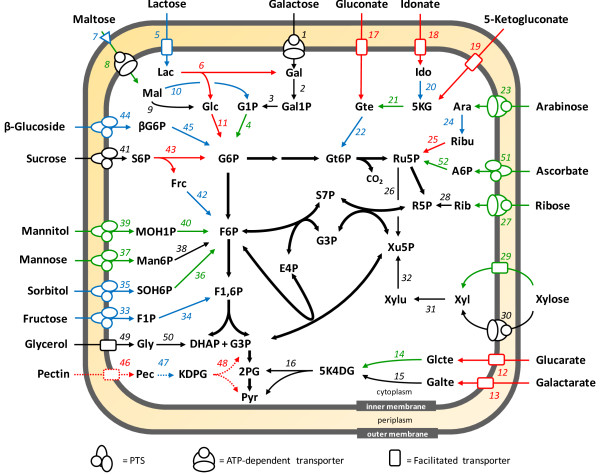
***A. succinogenes *uptake and degradation pathways for sugars other than glucose**. See additional file 2: Table S1 for more details. Arrow and number colors: black, product function assumed; green, putative function assumed; blue, probable function assumed; red, possible function assumed. Bold arrows, glycolytic and pentose phosphate pathways as described in Figure 2 and additional file 2: Table S3; Outer-membrane triangle, maltoporin. Numbers refer to enzymes or transporters described in additional file 2: Table S1. Metabolites: 5K4DG, 5-dehydro-4-deoxy-D-glucarate; 5 KG, 5-ketogluconate; Ara, L-arabinose; A6P, ascorbate-6-phosphate; DHAP, dihydroxyacetone phosphate; F1P, fructose-1-phosphate; Frc, fructose; Gal, galactose; Gal1P, galactose-1-phosphate; Galte, galactarate; Glcte, glucarate; Gly, glycerol; Gte, gluconate; Gt6P, gluconate-6-phosphate; G1P, glucose-1-phosphate; βG6P, β-glucoside-6-phosphate; Gt6P, gluconate-6-phosphate; Ido, idonate; KDPG, 2-keto-3-deoxy-6-phosphogluconate; Lac, lactose; Mal, maltose; Man6P, mannose-6-phosphate; MOH1P, mannitol-1-phosphate; Pec, pectin; Rib, ribose; Ribu, ribulose; S6P, sucrose-6-phosphate; SOH6P, sorbitol-1-phosphate; Xyl, xylose; Xylu, xylulose. Other abbreviations are as in Figure 2.

*A. succinogenes *grows on a wide variety of industrially relevant sugars including glucose, fructose, xylose, L-arabinose, mannose, and sucrose [1]. Figure 3 and additional file 2: Table S1 summarize the sugar uptake and degradation pathways that were identified in the genome sequence. Phosphotransferase systems (PTS) were identified for glucose, fructose, mannose, sorbitol, mannitol, sucrose, and β-glucosides (e.g., amygdalin, aesulin, arbutin, cellobiose, gentiobiose, and salicin). Galactose, maltose, arabinose, ribose, and xylose are taken up by ATP-dependent transporters. Xylose can also be transported by a H^+^-symport mechanism involving a separate transporter (Asuc_0496). After transport, L-arabinose is converted to ribulose by arabinose isomerase (Asuc_0494) and then phosphorylated by ribulokinase. Interestingly, the enzyme believed to be ribulokinase (Asuc_0493), based on its location in the arabinose operon (Asuc_0489-94), is only 20% identical to the *E. coli *enzyme. Facilitated transporters were also identified for lactose, gluconate, idonate, 5-ketogluconate, glucarate, galactarate, pectin or pectate, and glycerol. *A. succinogenes *was recently shown to grow on glycerol as its sole carbon source with dimethyl sulfoxide (DMSO) or nitrate as the terminal electron acceptor (Schindler and Vieille, manuscript in preparation). Accordingly, the *A. succinogenes *genome encodes a glycerol uptake facilitator (Asuc_1603), a glycerol kinase (Asuc_1604), and an anaerobic glycerol-3-phosphate dehydrogenase (Asuc_0205-3). It also encodes a DMSO reductase (Asuc_1524-1521), a periplasmic nitrate reductase (NapFDAGHBC, Asuc_2040-35) and a periplasmic nitrite reductase (NrfABCDEFG, Asuc_0704-11). In contrast, *M. succiniciproducens *contains only a truncated homolog of Asuc_1521 and lacks the other ORFs for the DMSO reductase complex.

Many sugar transport and degradation proteins have associated regulatory proteins (e.g., for L-arabinose, maltose, ribose, xylose, lactose, sorbitol, mannitol, glucarate and galactarate, and glycerol; additional file 2: Table S1). The ORFs putatively encoding sugar transport and degradation pathways encompass all the sugars *A. succinogenes *is known to use, except arabitol [1,6]. Homologs of the *E. coli *arabitol transporter and arabitol dehydrogenase were not found in the *A. succinogenes *genome. The genome also encodes transporters and degradation pathways for carbon sources *A. succinogenes *was not known to metabolize (e.g., ascorbate, pectin, glucarate, and galactarate). In the conditions tested, though, we were able to detect growth on galactarate and ascorbate only. Growth on ascorbate was surprising because the ascorbate PTS transporter encoded by Asuc_0235-40, appears to be missing a component. It is not clear whether this missing component is encoded elsewhere or whether another PTS system shares some specificity for ascorbate. The absence of growth on pectin was surprising, since *A. succinogenes *contains three separate operons encoding a full pectin degradation pathway (Asuc_0145-58, 0366-74, and 1467-75). Three of *A. succinogenes*'s ten possible tripartite ATP-independent periplasmic transporters (Asuc_0146-48, 0156-58, and 0366-68) are found in these operons. Several other operons appear to have a role in sugar transport and degradation, but the identity of the sugars is unknown. For example, one of the four proteins that show similarity to an idonate, a gluconate, or a 5-ketogluconate transporter is in an operon that encodes enzymes to degrade an unidentified sugar (Asuc_0119-30). Also, Asuc_0585-8 encodes a fructose-like PTS system and a protein that might be a sugar kinase.

### Explanation for lack of pathogenicity

The natural Pasteurellaceae ecology is in association with a host [52]. The host is usually mammalian, with a few exceptions [7], such as *P. multocida *colonizing birds [52] and possibly even amoebas [53]. Most Pasteurellaceae can be isolated from healthy hosts and are considered part of the normal flora. However, in circumstances such as host stress, many Pasteurellaceae cause disease and are considered opportunistic pathogens. Most Pasteurellaceae are isolated from the respiratory tract and cause pulmonary diseases [52]. Others have been isolated from the oral cavity (e.g., *A. actinomycetemcomitans*, which causes periodontitis), the genital tract (e.g., *H. ducreyi*, which causes sexually-transmitted chancroid), and bovine rumen (e.g., *A. lignieresii*, which causes wooden tongue) [7]. Virulence is an undesirable trait for an industrial organism, and the relatedness of *A. succinogenes *and *M. succiniciproducens *to several pathogens cannot be ignored. With no reports of disease caused by the succinogens, their genome sequences are a convenient and logical starting point to assess their potential for lack of pathogenicity.

Many Pasteurellaceae virulence factors have been characterized. We manually compiled a list of 341 Pasteurellaceae virulence proteins (with some functional redundancy), including and expanding on those compiled by Challacombe and Inzana [54]. We then aligned their sequences against the *A. succinogenes *and *M. succiniciproducens *protein databases (additional file 3: Table S6). Our comparison focuses on gene products having functions in toxin production, synthesis of cell surface structures, and iron uptake (additional file 3: Table S6). We excluded from the comparison virulence factors in the categories of amino acid transporters, purine and pyrimidine biosynthetic enzymes, and enzymes for anaerobic metabolism. While these metabolic activities may affect host health [55-57], they are also necessary for nonvirulent processes. The major findings from our alignments are summarized in this section with more details available in additional file 4: Supplementary text. Raw data from the alignments are reported in additional file 3: Table S6.

#### Toxins

Repeat toxins (RTX), including leukotoxins, are produced by many Pasteurellaceae, including *A. pleuropneumoniae *[58], *A. actinomycetemcomitans *[59], *M. haemolytica *[60], and *P. multocida *[61]. *A. actinomycetemcomitans *uses a cytolethal distending toxin that is encoded near a characteristic virulence-associated region [62]. Neither succinogen genome sequence encodes the components necessary to produce leukotoxin or cytolethal distending toxin. Additionally, neither succinogen encodes a homolog to the *H. ducreyi *hemolysin [63].

#### Cell surface structures

Cell surface virulence factors used by pathogenic Pasteurellaceae include pili, adhesins, lipopolysaccharide (LPS), and capsules. Adherence to respiratory epithelial cells is the first colonization stage by respiratory Pasteurellaceae pathogens. It involves a number of cell surface mechanisms [64]. Both succinogens have possible homologs to OapA and B and all components of a type IV pilus (*pilABCD*), which is involved in host surface binding in *H. influenzae *[65,66]. Because type IV pili are also part of the *H. influenzae *competence regulon [31], and because *A. succinogenes *was recently shown to be naturally competent (Joshi et al., manuscript in preparation), type IV pili might not be related to virulence in the succinogens. *M. succiniciproducens *has probable homologs of the *A. actinomycetemcomitans *pili needed for tight adherence (*flp *and *tad *loci) [67], whereas *A. succinogenes *does not. Both succinogens have several large ORFs that could encode HMW adhesins, including gene clusters that may be involved in hemagglutinin production (Asuc_1006-12 and MS1162-9). However, Asuc_1006 and 1008 have internal frame shifts. It is currently not known whether either succinogen makes an adhesin. It is also possible that the succinogens use this feature for survival in a competitive rumen environment, rather than to cause disease.

Nontypeable *H. influenzae *strains are able to evade host immune defenses by incorporating host sialic acid and choline into their LPS, thereby mimicking host cell surfaces [68,69]. The succinogens' genome sequences contain many genes involved in LPS synthesis and modification but key genes for choline and sialic acid incorporation are not present. None of these genes contain variable number tandem repeats, suggesting that the succinogens are not capable of LPS phase variation.

*A. succinogenes *has several LPS glycosyltransferases not found in *M. succiniciproducens *(e.g., Asuc_0524, 1375), suggesting that its LPS could be more complex. The two succinogens' LPSs might also differ in sugar composition. *A. succinogenes *is one of only four Pasteurellaceae that encodes the L-rhamnose synthesis pathway. L-rhamnose is a common component of the LPS O-antigen [70,71], and the *A. succinogenes *L-rhamnose biosynthetic pathway (Asuc_0826-32) is encoded just downstream of the LPS biosynthesis genes (Asuc_0821-24). Because LPS O-antigens are mostly studied in pathogenic bacteria, it is unclear how often non-pathogenic bacteria contain rhamnose in their LPS. For this reason, the possible presence of rhamnose in *A. succinogenes *LPS is by no means indicative of a virulence trait. In contrast, *M. succiniciproducens *encodes proteins likely involved in L-rhamnose transport (RhaT, MS2326) and catabolism (RhaBAD, MS2327-29), as well as the L-rhamnose-dependent regulators RhaS (MS2322) and RhaR (MS2323). None of these genes are found in *A. succinogenes*. Thus, the two succinogens have evolved completely different L-rhamnose pathways--a biosynthetic one in *A. succinogenes *and a catabolic one in *M. succiniciproducens*.

*P. multocida, A. pleuropneumoniae*, *M. haemolytica*, and typeable *H. influenzae *produce a capsule that is important for virulence [72-76]. Both succinogens have possible homologs to, at most, two of the four capsule biosynthesis and export proteins, suggesting that they are not capsulated bacteria. However, non-typeable *H. influenzae *are non-capsulated but they are still virulent.

#### Iron uptake mechanisms

Iron acquisition is a common trait in most bacteria, making it difficult to associate iron uptake with virulence. Nonetheless, some insight can be gained from the form of iron transported. Some pathogenic Pasteurellaceae can use the mammalian iron sources, transferrin and hemoglobin [72,77], but the succinogens have possible homologs to only a few of the proteins required for transferrin, heme/hemopexin, or hemoglobin uptake. *A. succinogenes *does not have homologs to the hemin receptor HemR or to the heme utilization protein Hup [66], while *M. succiniciproducens *has possible homologs of each. Still, BLAST searches show that both succinogens can assimilate other forms of iron, including iron bound by various siderophores and that they contain the heme biosynthetic pathway from L-glutamate [78] (additional file 3: Table S6). In both succinogens the potential hemagglutinin production system mentioned above is encoded alongside genes involved in ferrous iron transport, including *feoAB*. FeoA and B are not encoded in any sequenced Pasteurellaceae other than the succinogens and *A. minor *NM305, but they have been implicated in more distantly related bacteria in virulence and colonization of mammalian intestines [79,80]. This genetic region may be a worthwhile target for deletion, provided it does not contain essential genes for growth and succinate production.

#### Other virulence proteins

Both succinogens have a putative homolog to the inner membrane protein, ImpA, involved in autoaggregation [59]. Some Pasteurellaceae have a urease, which is a known virulence factor of gastroduodenal and urinary tract pathogens [81], but the succinogens have no urease homologs, and *A. succinogenes *tested negative for urease activity [1]. There is also no homolog in either succinogen genome sequence to the *H. influenzae *Iga protease, which cleaves immunoglobulin A1, helping *H. influenzae *avoid host defenses at mucosal surfaces [82,83].

We want to stress that nonpathogenicity cannot be concluded from the analysis of a genome sequence. Most Pasteurellaceae species cause respiratory diseases. The virulence factors associated with a hypothetical rumen succinogen-caused disease would likely be different. For example, the FeoAB iron uptake system, which is important for the virulence of some intestinal pathogens, is unique to the succinogens among the thirty-two partially and fully sequenced Pasteurellaceae, with *A. minor *NM305 (part of the pig respiratory tract normal flora) an exception. This system, though, could also be important for a commensal relationship with the mammalian host.

## Conclusions

Sequencing of the *A. succinogenes *genome confirms many of our earlier results based on growth experiments, enzyme assays, and metabolic flux studies [6,9,34,35]. For example, *A. succinogenes *lacks a complete TCA cycle as well as a glyoxylate pathway, and PEP carboxykinase is the only PEP-carboxylating enzyme in this organism. The genes missing in the glutamate, cysteine, and methionine biosynthetic pathways represent possible positive markers that can be used in genetic engineering strategies. The fact that the *A. succinogenes *genome encodes uptake and degradation pathways for a variety of carbohydrates reflects the variety of carbohydrate substrates available in the rumen, *A. succinogenes*'s natural habitat. It also suggests that many different carbon sources can be used as feedstock for succinate production by *A. succinogenes*. The abundance of USS repeats in *A. succinogenes *and the possible presence of the necessary machinery for natural competence suggested that *A. succinogenes *is naturally competent, a feature that was recently demonstrated in our laboratory. It is encouraging that the succinogens' genome sequences lack a considerable number of the virulence genes used by their relatives, and that there are no reports of disease caused by *A. succinogenes *or *M. succiniciproducens*. The lack of pathogenicity of these two succinogens is an exciting prospect not just for industrial purposes, but because comparisons with pathogenic Pasteurellaceae could lead to a better understanding of Pasteurellaceae virulence.

## Authors' contributions

JBM participated in the design of the study, prepared the genomic DNA for sequencing, supervised the manual annotation, performed most genomic comparisons, and drafted most of the manuscript. JBM, ML, BS, KB, VH, and CV participated in the manual annotation of the genome. BS determined the vitamin requirements, tested growth on various carbon sources, and helped draft the manuscript. AAM and SS participated in genomic comparisons and helped draft the manuscript. JFC contributed to the section on virulence traits. SRL, AC, and ALL were responsible for assembling and finishing the genome sequence. CV conceived the study, participated in its design and coordination, and helped draft the manuscript. All authors read and approved the final manuscript.

## Supplementary Material

Additional file 1 **Figures S1 to S6**. **Figure S1: Phylogenetic tree of representative Pasteurellaceae with complete genomes based on 16 S RNA sequences**. 16 S rRNA phylogeny was determined using the Michigan State University Ribosomal Database Project tools [19]. **Figure S2: Hierarchical clusterings of Pasteurellaceae species according to COGS, PFAM, Enzymes, and TIGRfam classifications**. Hierarchical clustering of Pasteurellaceae genomes was done according to COG, Pfam, Enzyme, and TIGRfam functional profiles at the JGI's Integrated Microbial Genomes website [21]. The four functional profile clustering approaches place the two succinogens in a clade separate from other Pasteurellaceae. **Figure S3: NUCmer and PROmer alignments of *A. succinogenes *and *M. succiniciproducens*, *P. multocida*, and *A. pleuropneumoniae *L20**. Synteny plots of the whole-genome alignments of *A. succinogenes *and *M. succiniciproducens*, *A. succinogenes *and *P. multocida*, and *A. succinogenes *and *A. pleuropneumoniae *L20 at the nucleotide level (NUCmer) and at the protein level (PROmer). Alignments were performed using the mummer software package [15]. These plots give overviews of the rearrangements that have taken place at the genome level between two bacterial species. Red lines from the bottom left to upper right indicate conservation of nucleotide (NUCmer) or protein (PROmer) sequence, reading in the same direction in both species. Blue lines from upper left to lower right indicate sequence conservation but with sequence inversion between the two species. NUCmer and PROmer comparisons of *A. succinogenes *with *H. influenzae *KW20, *H. influenzae *028NP, *H. somnus*, *H. ducreyi*, and *A. pleuropneumoniae *JL03 were also performed, but are not shown in this Figure. The NUCmer plots show little to no conservation of genome structure at the nucleotide level between *A. succinogenes *and any other *Pasteurellaceae*. PROmer plots reveal that *A. succinogenes *and *M. succiniciproducens *are more related to each other than to other Pasteurellaceae. The PROmer plot of *A. succinogenes *vs. *M. succiniciproducens *shows that drastic changes in genome structure have occurred as *A. succinogenes *and *M. succiniciproducens *evolved divergently from their last common ancestor, indicating that the two succinogens are more distantly related than their functional traits would suggest. **Figure S4: Comparison of nucleotide frequencies in Pasteurellaceae uptake signal sequences**. Figure S4 shows nucleotide frequencies in the USSs of six representative Pasteurellaceae species containing either USS1 (*A. succinogenes*, *M. succiniciproducens*, *A. aphrophilus *NJ8700, and *H. somni *129PT) or USS2 (*A. pleuropneumoniae *L20 and *H. ducreyi *3500HP). USS 9-mer cores were counted and their surrounding sequences reported using a perl script. The output was pasted into a Microsoft Excel spreadsheet to calculate the frequency of each nucleotide occurring at each position, upstream and downstream of the USS core. Nucleotide frequencies in the USSs of sixteen more Pasteurellaceae species containing USS1 (*H. influenzae *Rd KW20, 028NP, PittEE, PittAA, PittGG, PittHH, PittII, 22.1-21, 22.4-21, 3655, R2846, 2866, and R3021*; P. multocida*; *A. actinomycetemcomitans*; and *H. somni 2336*) and four more Pasteurellaceae species containing USS2 (*A. pleuropneumoniae *JL03 and 4074, *M. haemolytica *PHL213, and *H. parasuis *29775) were also calculated, but are not shown here. These data are available upon request. **Figure S5: *A. succinogenes *has incomplete pathways for assimilatory sulfate reduction and methionine synthesis**. Four-digit numbers are Asuc_ORF (locus tags) numbers and are followed by E.C. numbers. Hyphenated locus tag numbers indicate that the enzyme is encoded by several successive genes. Reaction names: see additional file 2: Table S4. XH, reduced thioredoxin; X^+^, oxidized thioredoxin. Arrow and number colors: black, product function assumed; blue, probable function assumed; red, possible function assumed. Bold arrows indicate central metabolic pathways. Dotted arrows indicate that *A. succinogenes *is missing the gene for that function. **Figure S6: *A. succinogenes *has incomplete pathways for biotin, nicotinic acid, pantothenic acid, and pyridoxine synthesis**. Four-digit numbers are Asuc_ORF (locus tags) numbers and are followed by E.C. numbers. Hyphenated locus tag numbers indicate that the enzyme is encoded by several successive genes. Reaction names: see additional file 2: Table S4. Arrow and number colors: black, product function assumed; green, putative function assumed; blue, probable function assumed; red, possible function assumed. Bold arrows indicate central metabolic pathways. Gray dotted arrows indicate that *A. succinogenes *is missing the gene for that function. Metabolites: Alac, 2-acetolactate; AON, 8-amino-7-oxonoanoate; APP, 3-amino-2-oxopropyl phosphate; CoA, coenzyme A; Dbio, dethiobiotin; DCoA, dephospho-CoA; DhP, 2-dehydropantoate; DMB, 2,3-dihydroxy-3-methylbutanoate; dNAD^+^, deamido-NAD^+^; DON, 7,8-diaminononanoate; DXP, 1-deoxyxylulose-5-phosphate; Er4P, erythronate-4-phosphate; HPB, 2-oxo-3-hydroxy-4-phosphobutanoate; IAsp, iminoaspartate; MOB, 3-methyl-2-oxobutanoate; NRS, nicotinate ribonucleoside; NRT, nicotinate ribonucleotide; Pan, pantoate; PCA, pimeloyl-CoA; PHT, O-phospho-4-hydroxythreonine; Pim, pimelate; PNP, pyridoxine phosphate; Ppc, 4'-phosphopantothenoyl-cysteine; Ppt, 4'-phosphopantothenate; Ppth, 4'-phosphopantetheine; QNL, quinolinate. Other abbreviations are as in Figure 2. Click here for file

Additional file 2 **Tables S1 to S5**. **Table S1: *A. succinogenes *ORFs encoding sugar transporters and degradation pathways**. Table S1 lists all the *A. succinogenes *transporters, enzymes, and regulatory proteins potentially involved in sugar transport and assimilation, based on our manual annotation of the genome. Annotation criteria are described in the materials and methods section. The ORFs putatively encoding sugar transport and degradation pathways encompass all the sugars *A. succinogenes *is known to use, except arabitol. The *A. succinogenes *genome also encodes transporters and degradation pathways for carbon sources *A. succinogenes *does not metabolize (e.g., pectin). **Table S2: *A. succinogenes *homologs of *H. influenzae *competency proteins**. List of the *H. influenzae *competency genes and their *A. succinogenes *homologs, with the likeliness that the *A. succinogenes *homologs have the same function. *A. succinogenes *homologs are considered putative if they share 60-75% amino acid identity with the query sequence, probable if they share 40-59% amino acid identity with the query sequence, and possible if they share 25-39% amino acid identity with the query sequence. NA indicates that no suitable homolog was identified in *A. succinogenes *either due to insufficient alignment length (less than 25% of the query sequence length) or to no hits retrieved from the BLAST search. **Table S3: *A. succinogenes *ORFs encoding central metabolic enzymes**. List of *A. succinogenes *genes encoding enzymes of central metabolism with their locus names and EC numbers. Enzyme names are based on our manual annotation of the genome, using the criteria described in the materials and methods section. **Table S4: Partial biosynthetic pathways present in *A. succinogenes *for amino acids and vitamins required for growth**. Cysteine, glutamate, methionine, biotin, nicotinic acid, pantothenate, and pyridoxine are required for *A. succinogenes's *growth on defined medium. Table S4 lists the components of the cysteine, methionine, biotin, nicotinic acid, pantothenate, and pyridoxine biosynthetic pathways that are present in *A. succinogenes*. Enzyme names are based on our manual annotation of the genome, using the criteria described in the materials and methods section. This list confirms that *A. succinogenes *contains an incomplete assimilatory sulfate reduction pathway, but that it is able to synthesize cysteine from sulfide or thiosulfate. It also suggests that *A. succinogenes *is unable to synthesize Met from L-homocysteine. **Table S5: *A. succinogenes *dicarboxylate transporters**. *A. succinogenes *excretes large amounts of succinate as well as smaller quantities of fumarate, but the succinate and fumarate transporters are unknown. Table S5 lists the twelve possible anaerobic dicarboxylate transporters identified in *A. succinogenes*. Transporter names are based on our manual annotation of the genome, using the criteria described in the materials and methods section. Percent identity to experimentally characterized transporters is indicated in parentheses. Nine *A. succinogenes *transporters are similar to the tripartite ATP-independent periplasmic transporter (T.C. 2.A.56) encoded by *dctPQM *[47]. The other three are related to DcuA, B, and C (T.C. 2.A.13). DcuA, B, and C operate by exchanging an intracellular dicarboxylate (e.g., succinate) for an extracellular dicarboxylate (e.g., fumarate, malate, or aspartate). DcuA and B may also transport Na^+ ^in symport with the dicarboxylates to avoid dissipating the proton motive force [48]. Based on studies performed in *E. coli *and *H. influenzae *(see manuscript for references), Asuc_0142, 1999, and 1063 are likely candidates genes for dicarboxylate transport during fumarate respiration and succinate fermentation. Click here for file

Additional file 3 **Table S6**. **Table S6: *A. succinogenes *and *M. succiniciproducens *proteins showing similarity to known Pasteurellaceae virulence factors**. Table S6 is an extensive list of known Pasteurellaceae virulence factors and their top BLAST hits in the *A. succinogenes *and *M. succiniciproducens *genomes associated with the best BLAST hits in *A. succinogenes *and *M. succiniciproducens*. Virulence factors are listed by category: cell surface structures, iron acquisition, toxins, and other. Each virulence factor (i.e., query sequence) is identified by its protein name, accession number, PubMed identifier (PMID), source organism, function, and length. Each *A. succinogenes *or *M. succiniciproducens *hit is identified by its accession number, locus number (only for *A. succinogenes*), length, E value, alignment length, percent identity, percent similarity, alignment length in percent of the length of the hit, validity of the hit (i.e., hit and reason columns), and product name in GenBank. Click here for file

Additional file 4 **Explanation for lack of pathogenicity--extended discussion**. The supplementary text contains an extended discussion of alignment of Pasteurellaceae virulence factors against succinogens' genomes. The discussion touches on many of the negative results not reported in the main text. Click here for file
