# Supplementary material for: A genomic perspective on the potential of Actinobacillus succinogenes for industrial succinate production
Source: BMC Genomics. 2010 Nov 30;11:680. doi: 10.1186/1471-2164-11-680 (PMC3091790; doi:10.1186/1471-2164-11-680)

Figure S1. Phylogenetic tree of representative Pasteurellaceae with complete genomes based on 16S RNA sequences.

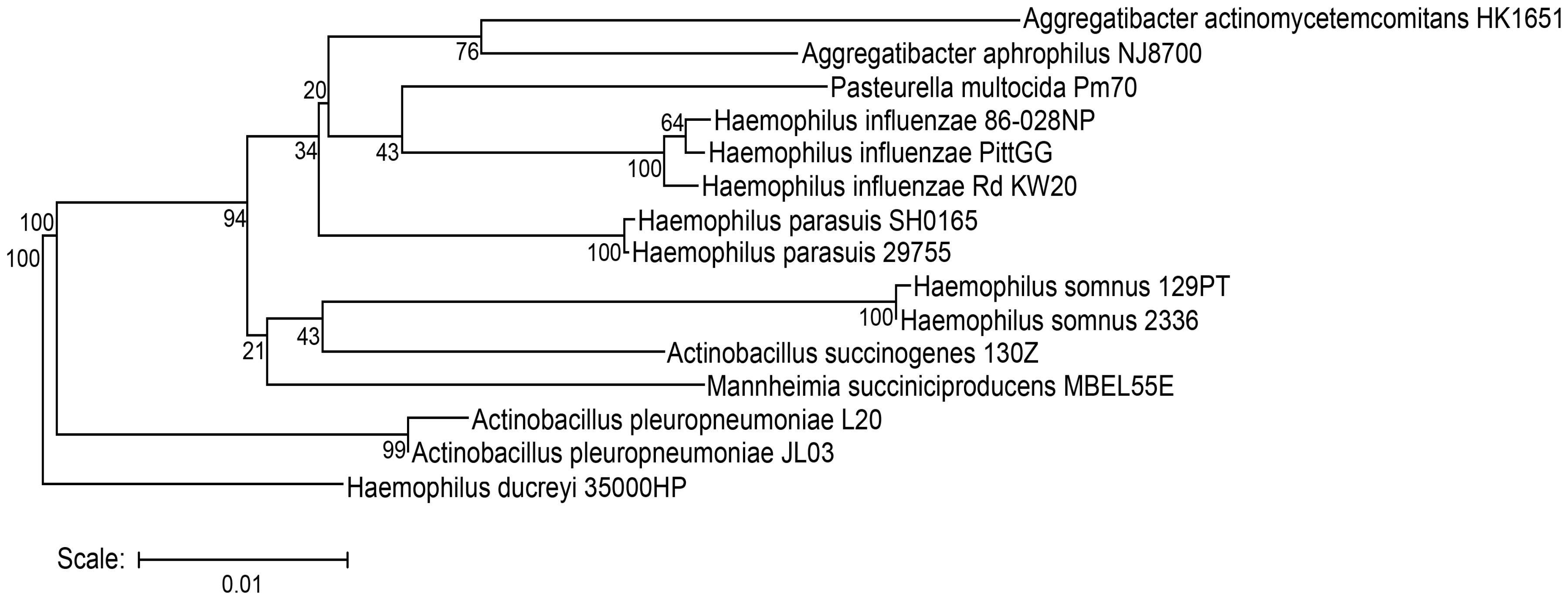

Figure S2. Hierarchical clusterings of Pasteurellaceae species according to COGS, PFAM, Enzymes, and TIGRfam classifications.

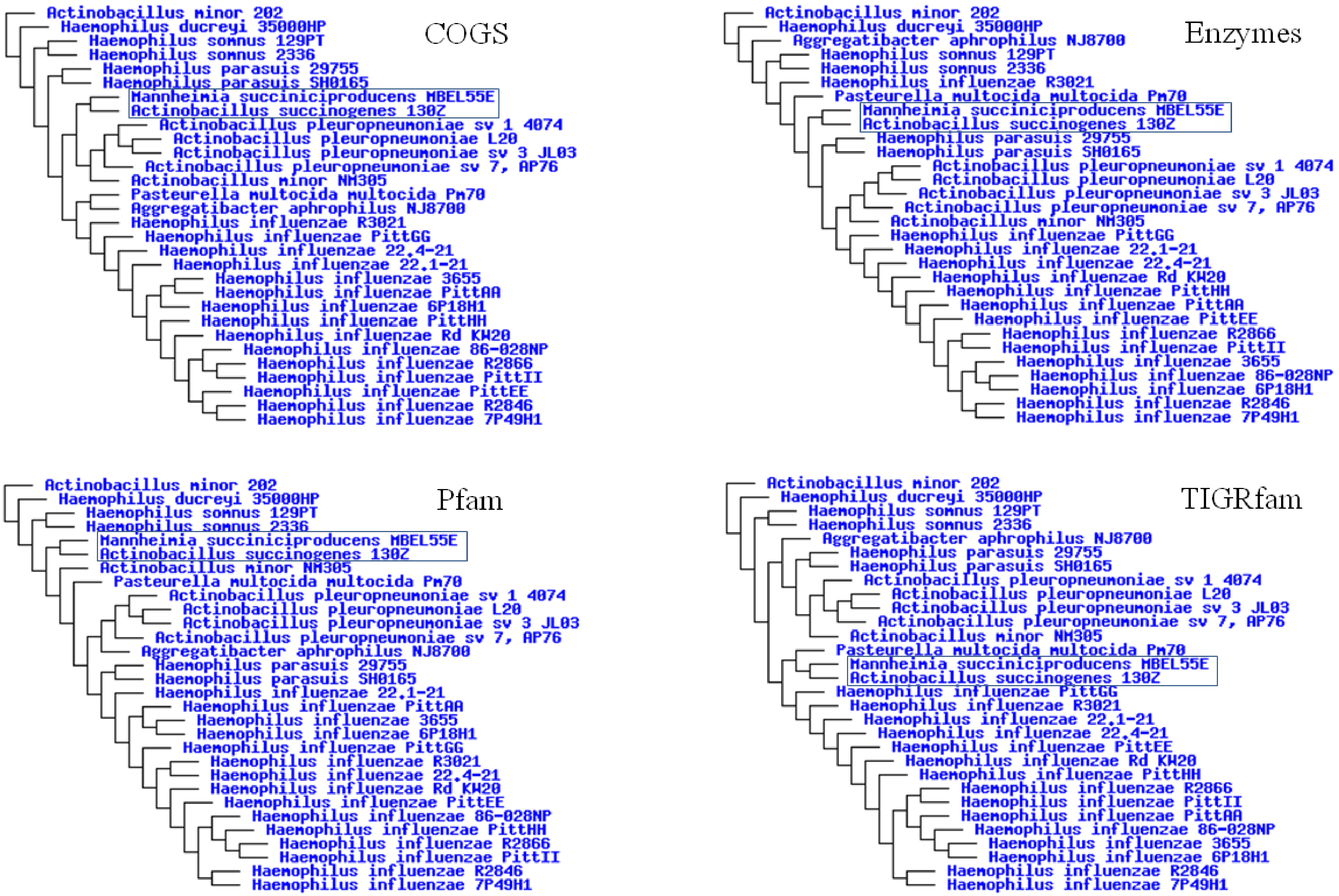

Figure S3. NUCmer and PROmer alignments of *A. succinogenes* and *M. succiniciproducens*, *P. multocida*, and *A. pleuropneumoniae* L20.

NUCmer alignments

PROmer alignments

A. succinogenes vs. *M. succiniciproducens*

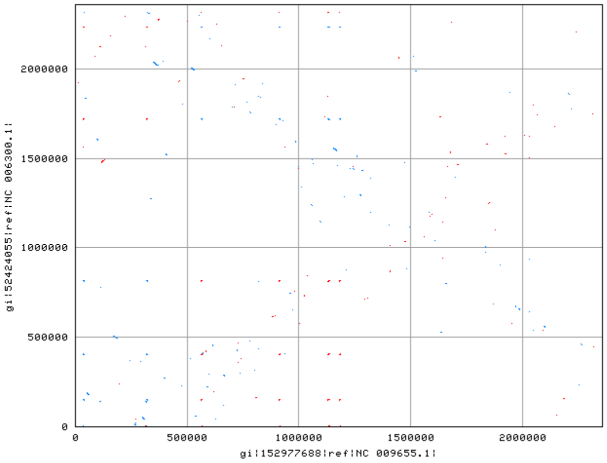

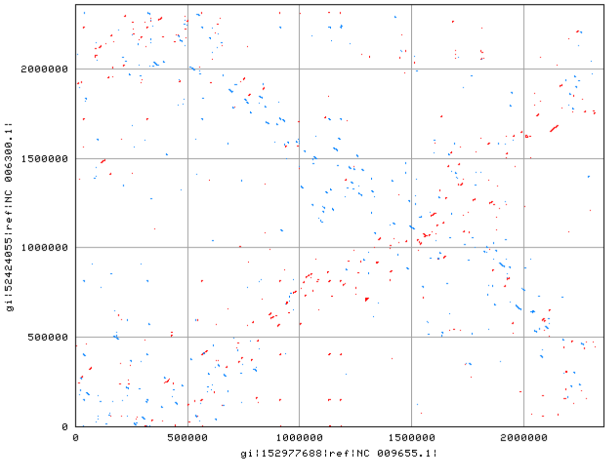

A. succinogenes vs. *P. multocida*

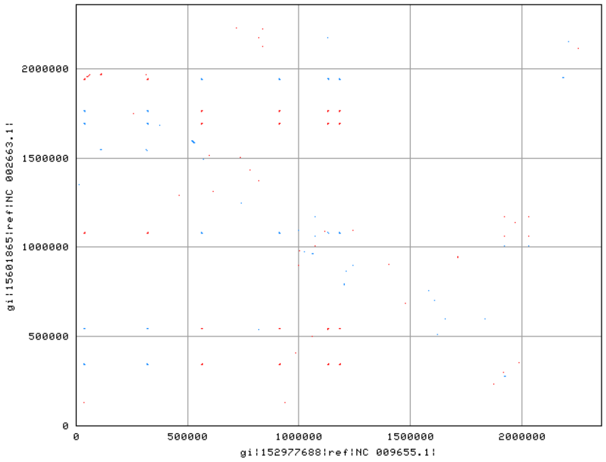

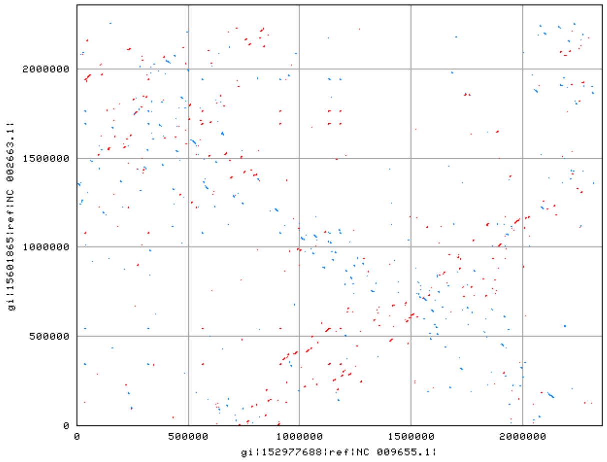

A. succinogenes vs. *A. pleuropneumoniae* L20

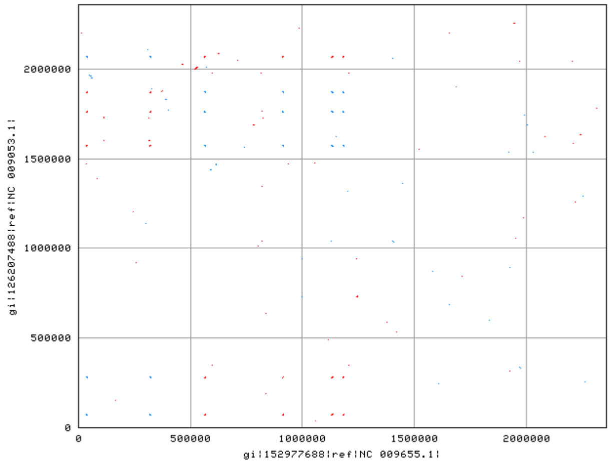

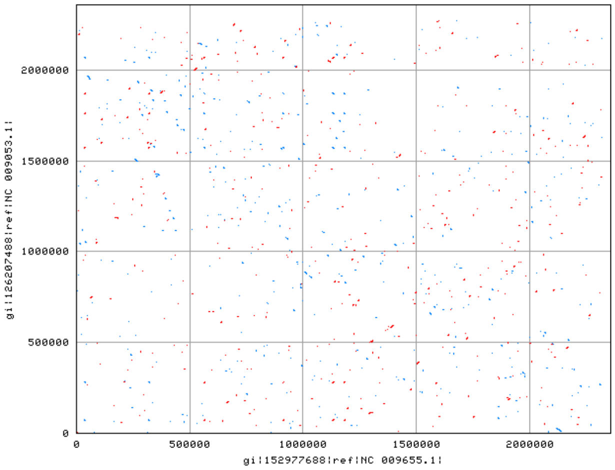

Figure S4: Comparison of nucleotide frequencies in Pasteurellaceae uptake signal sequences.

A. Uptake signal sequence version 1

Actinobacillus succinogenes

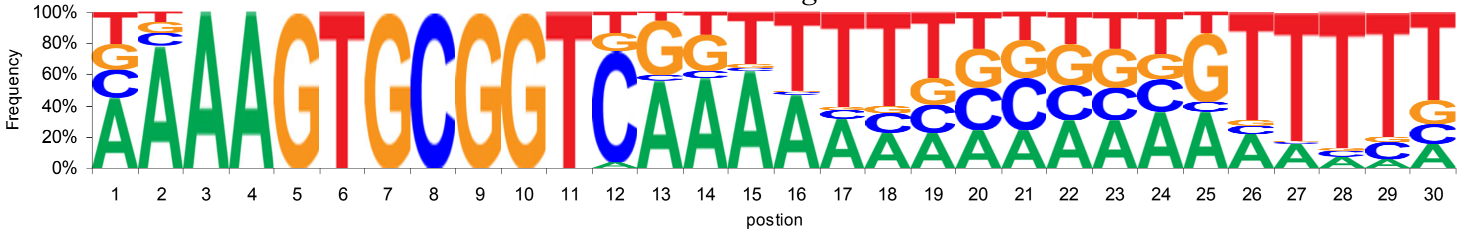

Mannheimia succiniciproducens

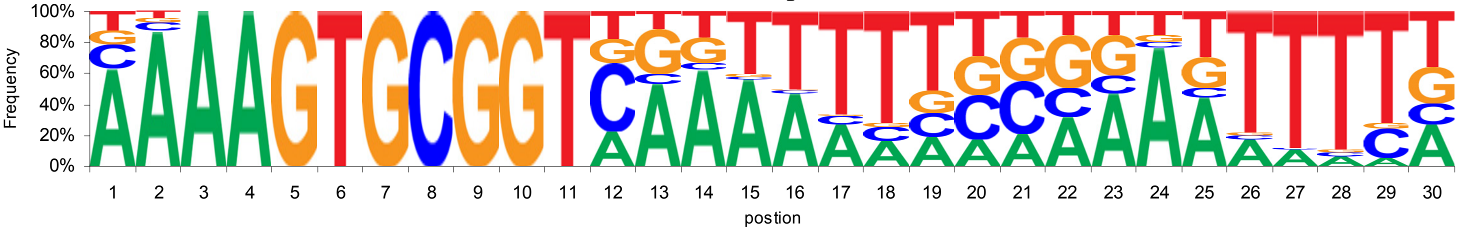

Aggregatibacter aphrophilus NJ8700

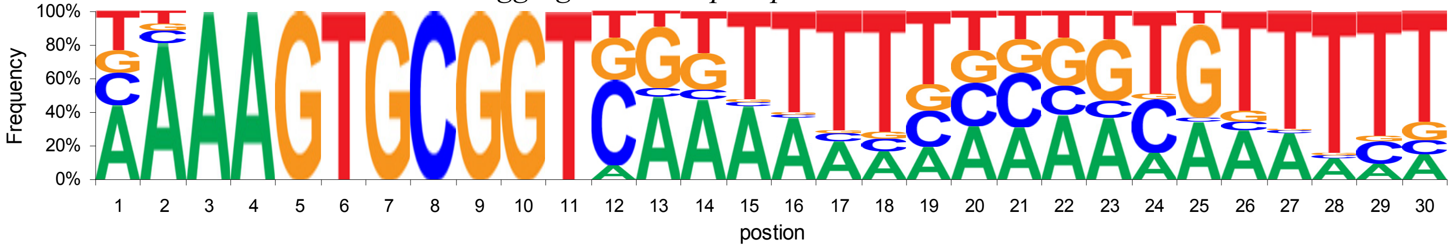

Histophilus somni 129PT

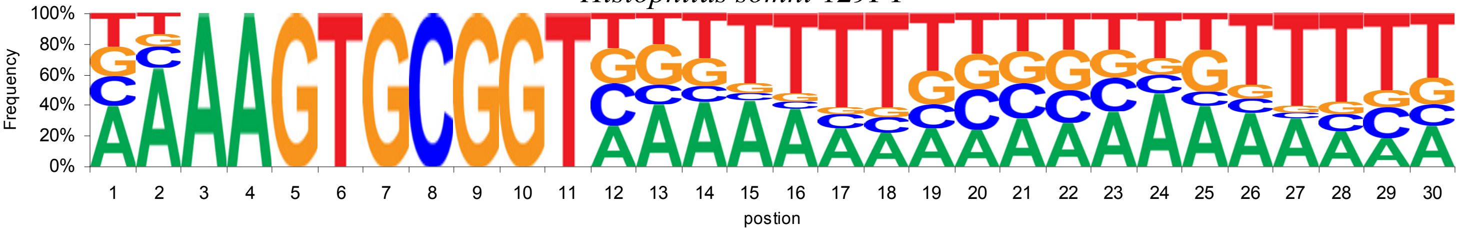

B. Uptake signal sequence version 2

Actinobacillus pleuropneumoniae L20

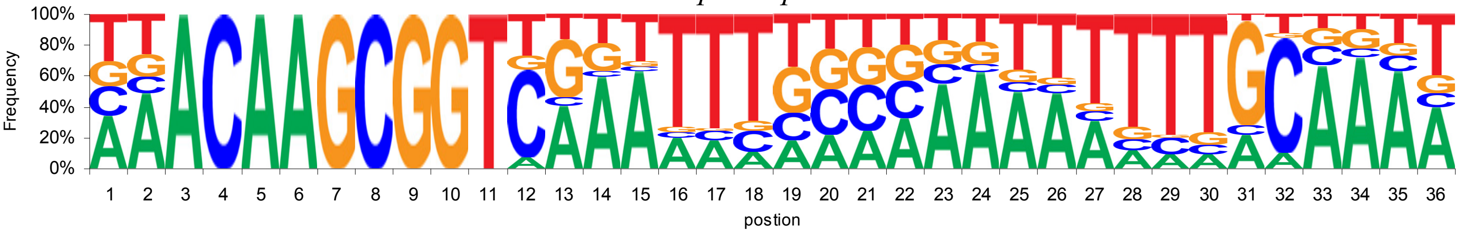

Haemophilus ducreyi 3500HP

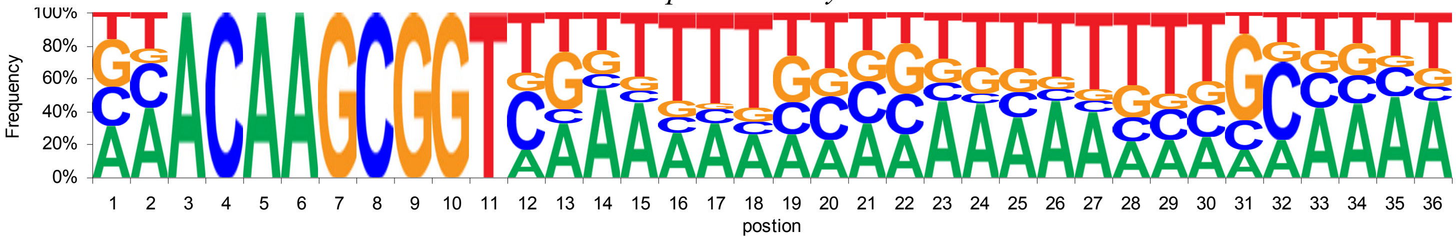

Figure S5: *A. succinogenes* has incomplete pathways for assimilatory sulfate reduction (A) and methionine synthesis (B).

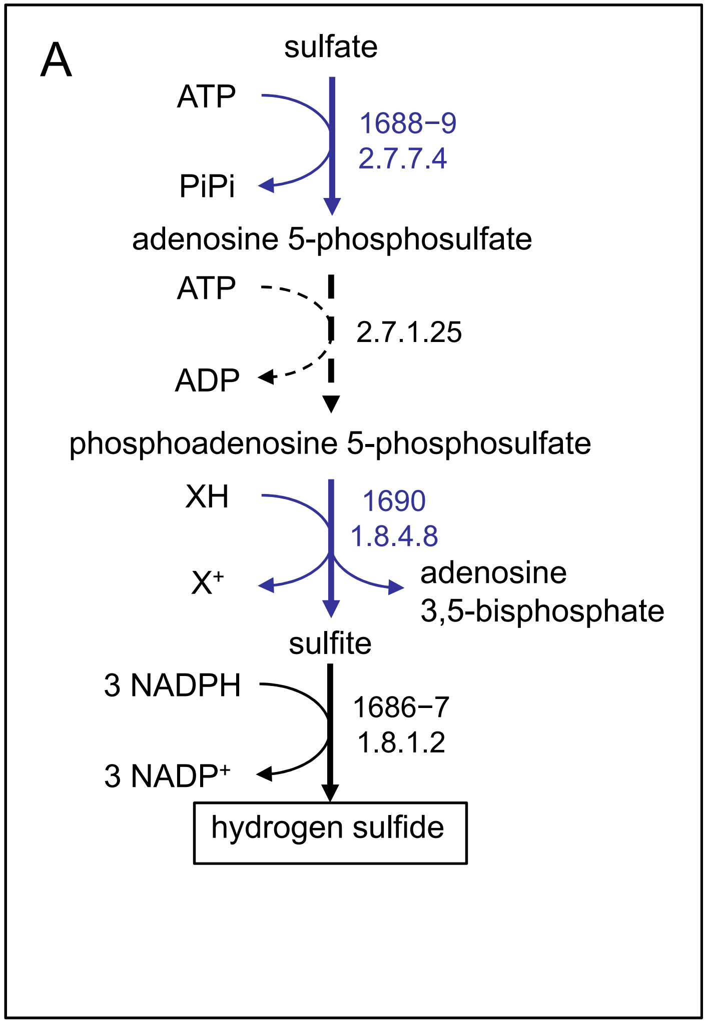

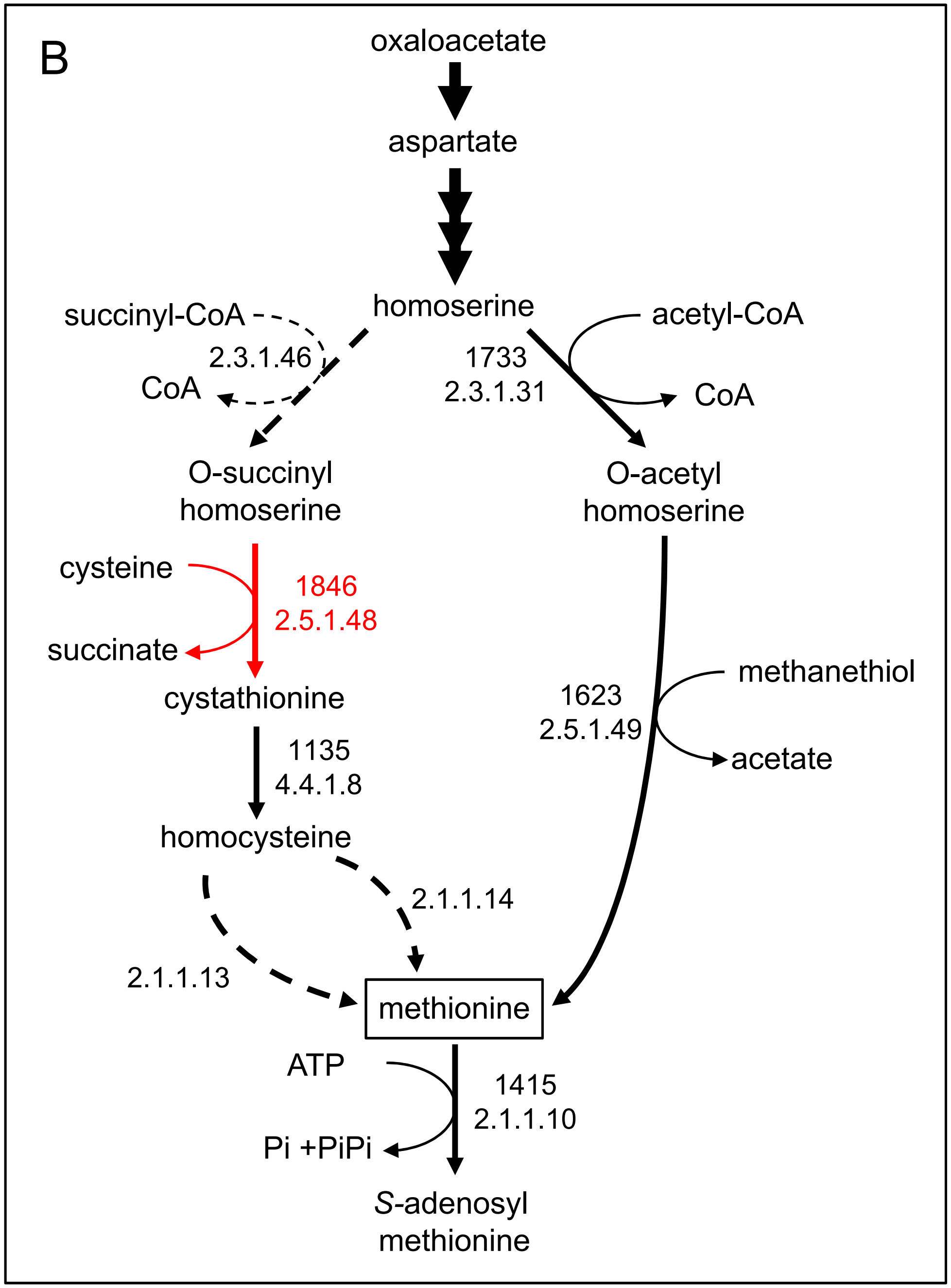

Figure S6. *A. succinogenes* has incomplete pathways for biotin, nicotinic acid, pantothenic acid, and pyridoxine synthesis.

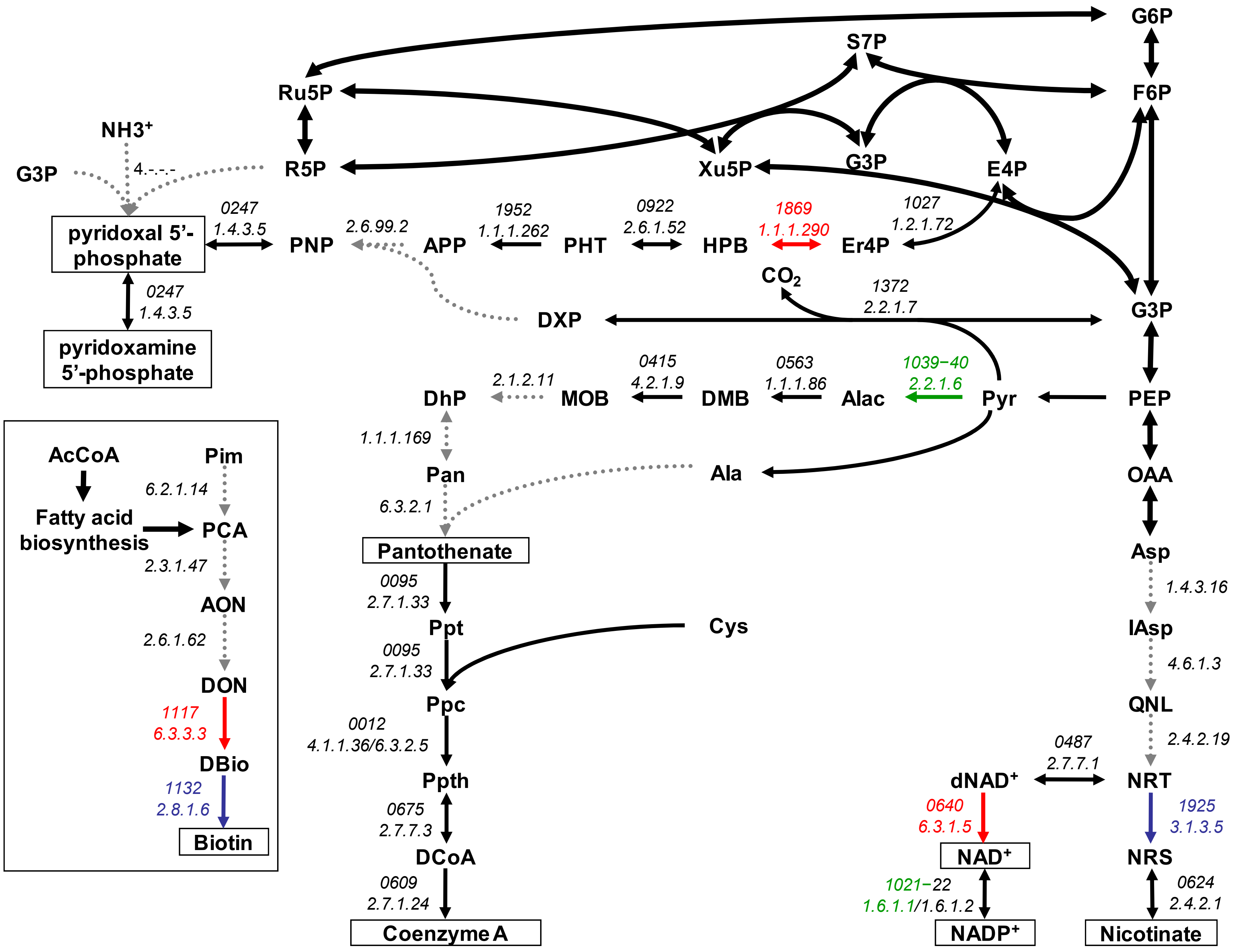

Supplement: Additional file 1 — Figures S1 to S6. Figure S1: Phylogenetic tree of representative Pasteurellaceae with complete genomes based on 16 S RNA sequences. 16 S rRNA phylogeny was determined using the Michigan State University Ribosomal Database Project tools [19]. Figure S2: Hierarchical clusterings of Pasteurellaceae species according to COGS, PFAM, Enzymes, and TIGRfam classifications. Hierarchical clustering of Pasteurellaceae genomes was done according to COG, Pfam, Enzyme, and TIGRfam functional profiles at the JGI's Integrated Microbial Genomes website [21]. The four functional profile clustering approaches place the two succinogens in a clade separate from other Pasteurellaceae. Figure S3: NUCmer and PROmer alignments of A. succinogenes and M. succiniciproducens, P. multocida, and A. pleuropneumoniae L20. Synteny plots of the whole-genome alignments of A. succinogenes and M. succiniciproducens, A. succinogenes and P. multocida, and A. succinogenes and A. pleuropneumoniae L20 at the nucleotide level (NUCmer) and at the protein level (PROmer). Alignments were performed using the mummer software package [15]. These plots give overviews of the rearrangements that have taken place at the genome level between two bacterial species. Red lines from the bottom left to upper right indicate conservation of nucleotide (NUCmer) or protein (PROmer) sequence, reading in the same direction in both species. Blue lines from upper left to lower right indicate sequence conservation but with sequence inversion between the two species. NUCmer and PROmer comparisons of A. succinogenes with H. influenzae KW20, H. influenzae 028NP, H. somnus, H. ducreyi, and A. pleuropneumoniae JL03 were also performed, but are not shown in this Figure. The NUCmer plots show little to no conservation of genome structure at the nucleotide level between A. succinogenes and any other Pasteurellaceae. PROmer plots reveal that A. succinogenes and M. succiniciproducens are more related to each other than to other Pasteurellaceae [file 1471-2164-11-680-S1.PDF]
