# Supplementary material for: A genomic perspective on the potential of Actinobacillus succinogenes for industrial succinate production
Source: BMC Genomics. 2010 Nov 30;11:680. doi: 10.1186/1471-2164-11-680 (PMC3091790; doi:10.1186/1471-2164-11-680)
Supplement: Additional file 2 — Tables S1 to S5. Table S1: A. succinogenes ORFs encoding sugar transporters and degradation pathways. Table S1 lists all the A. succinogenes transporters, enzymes, and regulatory proteins potentially involved in sugar transport and assimilation, based on our manual annotation of the genome. Annotation criteria are described in the materials and methods section. The ORFs putatively encoding sugar transport and degradation pathways encompass all the sugars A. succinogenes is known to use, except arabitol. The A. succinogenes genome also encodes transporters and degradation pathways for carbon sources A. succinogenes does not metabolize (e.g., pectin). Table S2: A. succinogenes homologs of H. influenzae competency proteins. List of the H. influenzae competency genes and their A. succinogenes homologs, with the likeliness that the A. succinogenes homologs have the same function. A. succinogenes homologs are considered putative if they share 60-75% amino acid identity with the query sequence, probable if they share 40-59% amino acid identity with the query sequence, and possible if they share 25-39% amino acid identity with the query sequence. NA indicates that no suitable homolog was identified in A. succinogenes either due to insufficient alignment length (less than 25% of the query sequence length) or to no hits retrieved from the BLAST search. Table S3: A. succinogenes ORFs encoding central metabolic enzymes. List of A. succinogenes genes encoding enzymes of central metabolism with their locus names and EC numbers. Enzyme names are based on our manual annotation of the genome, using the criteria described in the materials and methods section. Table S4: Partial biosynthetic pathways present in A. succinogenes for amino acids and vitamins required for growth. Cysteine, glutamate, methionine, biotin, nicotinic acid, pantothenate, and pyridoxine are required for A. succinogenes's growth on defined medium. Table S4 lists the components of the cysteine, methionine, biotin, [file 1471-2164-11-680-S2.PDF]

Table S1. *A. succinogenes* ORFs encoding sugar transporters and degradation pathways.

Asuc #	Function	E.C. or T.C. #	Fig. 3 #
	**Glucose**		
0994	PTS HPr protein		
0995	PTS EI component		
0996	PTS glucose-subfamily EIIA component		
0936−8	Putative mannose-specific PTS	4.A.6.1.1	37
0496	Sugar transport protein		
	Possible sugar kinase	2.7.1.2	11
	**Galactose**		
1896−8	ATP-dependent galactoside transporter	3.A.1.2.3	1
1901	Galactose mutarotase	5.1.3.-	
1900	Galactokinase	2.7.1.6	2
1899	Galactose-1-phosphate uridylyltransferase	2.7.7.12	3
1817	UDP-glucose 4-epimerase GalE	5.1.3.2	3
0049	UTP-glucose-1-phosphate uridylyltransferase GalU	2.7.7.9	3
0050	Putative phosphomannomutase (phosphoglucomutase?)	5.4.2.8/5.4.2.2	4
0051	Carbon storage regulator		
	**Lactose**		
1397	Probable lactose permease LacY	2.A.1.5.1	5
1398	Possible β-galactosidase LacZ	3.2.1.23	6
1396	Possible periplasmic binding protein/LacI transcriptional regulator		
	**Maltose**		
0314	Probable maltose regulon transcriptional regulator		
0315, 0321	Probable α-amylase	3.2.1.1	
0322	Probable maltoporin		7
0316−20, 0323	Putative ATP-dependent maltose transporter	3.A.1.1.1	8
0312	Amylomaltase	2.4.1.25	9
0313	Probable maltodextrin phosphorylase	2.4.1.1	10
1504	Possible glucokinase	2.7.1.2	11
	**Glucarate and galactarate**		
1584	Possible D-glucarate/galactonate transporter	2.A.1.14.1, 2.A.1.14.14	12, 13
1847	D-Glucarate dehydratase GudD	4.2.1.40	14
1852	Putative D-glucarate dehydratase	4.2.1.40	14
1853	Possible 2,5-diketo-D-gluconate reductase A	1.1.1.274	
1854	Possible gluconolactonase		
1855	D-galactarate dehydratase	4.2.1.42	15
1856	Putative carbohydrate diacid regulator		16
1857	Probable glycerate kinase GarK	2.7.1.-	16
1858	Tartronate semialdehyde reductase GarR	1.1.1.60	16
1859	Putative α-dehydro-β-deoxy-D-glucarate aldolase GarL	4.1.2.20	16
	**Gluconate, idonate, and 5-ketogluconate**		
1848	Possible L-idonate or gluconate transport protein	2.A.8.1.2	17, 18, 19
0889	Possible L-idonate or gluconate transport protein	2.A.8.1.2	17, 18, 19

0119	Possible L-idonate or gluconate transport protein	2.A.8.1.2	17, 18, 19
1157	Possible L-idonate or gluconate transport protein (IdnT)	2.A.8.1.2	17, 18, 19
1155	Probable L-idonate 5-dehydrogenase (IdnD)	1.1.1.-	20
1156	Putative gluconate 5-dehydrogenase (IdnO)	1.1.1.69	21
1154	Probable gluconokinase	2.7.1.12	22
	**Arabinose**		
0489−91	Putative high affinity ATP-dependent L-arabinose transporter (AraFGH)	3.A.1.2.2	23
0492	Probable arabinose operon regulatory protein		
0494	Probable L-arabinose isomerase	5.3.1.4	24
0493	Conserved hypothetical protein (possible xylulokinase)	2.7.1.17	25
0234	L-ribulose-5-phosphate 4-epimerase UlaF	5.1.3.4	26
	**Ribose**		
0194−7	Putative high affinity ATP-dependent ribose transporter (RbsDACB)	3.A.1.2.1	27
0198	Ribokinase	2.7.1.15	28
0199	Probable ribose operon repressor protein		
	**Xylose**		
0496	Putative low affinity D-xylose-proton symporter	2.A.1.1.3	29
0497−9	High affinity ATP-dependent xylose transporter (XylHGF)	3.A.1.2.4	30
0500	Xylose isomerase	5.3.1.5	31
0501	Xylulose kinase	2.7.1.17	32
0052	Probable L-xylulokinase		
0495	Putative xylose operon regulatory protein		
	**Fructose**		
0081−3	Possible/probable high affinity ATP-dependent allose/ribose transporter		
0085, 87	Probable fructose-specific PTS (FruBA)	4.A.2.1.1	33
0086	Probable 1-phosphofructokinase	2.7.1.56	34
	**Sorbitol**		
0434−6	Probable glucitol/sorbitol-specific PTS	4.A.4.1.1	35
0437	Putative sorbitol-6-phosphate 2-dehydrogenase	1.1.1.140	36
0439	Probable glucitol operon activator		
0440	Putative glucitol operon repressor		
	**Mannose**		
0936−8	Putative mannose-specific PTS	4.A.6.1.1	37
0934	Mannose-6-phosphate isomerase, class I	5.3.1.8	38
	**Mannitol**		
0455	Putative mannitol-specific PTS	4.A.2.1.2	39
0456	Putative mannitol-1-phosphate-5-dehydrogenase	1.1.1.17	40
0457	Possible mannitol repressor protein MtlR		
	**Sucrose**		
0914	Sucrose-specific PTS EIIBC	4.A.1.2.9	41
1828	Probable ATP-dependent fructokinase	2.7.1.4	42
	Possible sucrose hydrolase	3.2.1.26	43

	**β-glucosides**		
0975	Probable β-glucoside-specific PTS, IIABC component	4.A.1.2.2	44
0973	Probable 6-phospho-β-glucosidase	3.2.1.86	45
	**Pectin**		
1467	Putative oligogalacturonide lyase	4.2.2.6	47
1468	Conserved hypothetical Na^+ symporter		46
1469	Probable exopolygalacturonate lyase	4.2.2.9	47
1470	Probable pectin degradation protein		47
1471	Possible 2-keto-4-hydroxyglutarate aldolase (multifunctional)	4.1.2.14, 4.1.3.16	48
1472	Probable 2-dehydro-3-deoxygluconokinase	2.7.1.45	47
1473	Conserved hypothetical ribose/galactose isomerase (5-keto 4-deoxyuronate isomerase, based on operon)	5.3.1.17?	47
1474	2-Deoxy-D-gluconate 3-dehydrogenase	1.1.1.125	47
1475	Putative pectin degradation repressor protein KdgR		
0145	Putative glucuronate/galacturonate isomerase UxaC	5.3.1.12	47
0146−8	Tripartite ATP-independent periplasmic transporter		46
0366−8	Tripartite ATP-independent periplasmic transporter		46
0149	Possible α-glucosidase		
0150	Probable transcriptional hexuronate regulatory protein UxuR		
0372	Possible transcriptional galacturonate regulator		
0151	Mannonate dehydratase UxuA	4.2.1.8	47
0370	Mannonate dehydratase	4.2.1.8	47
0371	Possible mannoate oxidoreductase, NAD-dependent	1.1.1.57	
0152	Possible multifunctional 2-keto-3-deoxygluconate 6-phosphate aldolase and 2-keto-4-hydroxyglutarate aldolase and oxaloacetate decarboxylase	4.1.2.14, 4.1.3.16	48
0374	Possible multifunctional 2-keto-3-deoxygluconate 6-phosphate aldolase, 2-keto-4-hydroxyglutarate aldolase, and oxaloacetate decarboxylase (100% identical to 0152 nt seq)	4.1.2.14, 4.1.3.16	48
0153	Probable ketodeoxyglucokinase	2.7.1.45	47
0373	Probable ketodeoxyglucokinase (99% identical to 0153 nt seq)	2.7.1.45	47
0154	Putative altronate dehydratase UxaA	4.2.1.7	47
0155	Putative altronate oxidoreductase UxaB	1.1.1.58	47
0156−8	Tripartite ATP-independent periplasmic transporter		46
	**Glycerol**		
1603, 408	Glycerol transporter		49
1604	Glycerol kinase	2.7.1.30	50
0169	Probable Glycerol kinase	2.7.1.30	50
0092	Putative glycerol-3-phosphate regulon repressor		
0203−5	Glycerol-3 phosphate dehydrogenase (GlpABC)	1.1.1.8	50
	**Ascorbate**		
0238−9	Probable/putative partial ascorbate PTS?		
0237	Putative L-ascorbate 6-phosphate lactonase UlaG	3.1.1.-	
0240	Probable 3-keto-L-gulonate 6-phosphate decarboxylase UlaD	4.1.1.85	
0235	Probable L-xylulose 5-phosphate 3-epimerase UlaE	5.1.3.22	
0236	Probable DNA-binding transcriptional dual regulator of ascorbate operon, UlaR		

	Unknown
0585−8	PTS and kinase (galactitol or tagatose)
1167	Possible ketodeoxyglucokinase
1168	2-keto-D-gluconate reductase
1166	Transcriptional repressor
0120	Possible L-fuculose-1-phosphate aldolase (58% identical to YgbL)
0121	Sugar aldolase
0122	Probably related (similar to YgbK)
0123	71% identical to YgbJ
0124	Transcriptional regulator of sugar metabolism (53% identical to Ygbi)
0126−130	Sugar transport and phosphorylation
0131	Possible manno(fructo)/allose kinase

Table S2. *A. succinogenes* homologs of *H. influenzae* competency proteins.

[a] Putative, 60–75% amino acid identity; Probable, 40–59% amino acid identity; Possible, 25–39% amino acid identity; NA, no suitable homolog identified in *A. succinogenes* either due to insufficient length of alignment (less than 25% of the query sequence length) or to no hits retrieved from the BLAST search.

H. influenzae ORF #	***H. influenzae* product description**	Asuc_#	*A. succinogenes* modified product description[a]
HI0957	Catabolite gene activator (cAMP receptor protein)	0008	Putative
HI0061	Recombination protein	1729	Probable
HI1008	hypothetical protein HI1008 ComE1	1873	Putative
HI0435	Competence protein E	0688	Putative
HI0436	Competence protein D	0689	Possible
HI0437	Competence protein C	0690	Possible
HI0438	Competence protein B0	0691	Possible
HI0439	Competence protein A	0692	Possible
HI0365	Hypothetical protein HI0365	2030	Function
HI0296	Type 4 prepilin-like protein specific leader peptidase	0610	Possible
HI0297	Protein transport protein	0611	Probable
HI0298	Protein transport protein	0612	Putative
HI0299	Prepilin peptidase-dependent protein D	0613	Putative
HI0659	Uncharacterized HTH-type transcriptional regulator	0025	Possible
HI0660	Hypothetical, pilin-like protein HI0660	NA	NA (No hits)
HI0938	Hypothetical protein HI0938	1789	Probable
HI0939	Hypothetical protein HI0939	1788	Probable
HI0940	Hypothetical protein HI0940	1787	Possible
HI0941	Hypothetical protein HI0941	1786	Probable
HI0985	DNA processing chain A	0566	Probable
HI1117	Competence protein	0505	Function
HI0952	DNA repair protein RadC	0013	Putative
HI0250	Single-strand DNA-binding protein	1880	Putative
HI1182–3	DNA ligase	1188	Putative
HI1631	Hypothetical protein HI1631	0814	NA (length)
HI0601	DNA transformation protein Sxy	0283	Probable
HI0600	Recombinase A, RecA	0261	Function
HI1365	DNA topoisomerase I, TopA	1399	Function
HI0481	ATP synthase subunit alpha, AtpA	0328	Function
HI0846	Thiol:disulfide interchange protein, DsbA	0308	Putative

Table S3. *A. succinogenes* ORFs encoding central metabolic enzymes.

Asuc_#	Function	E.C. #
0994–6	PEP:sugar phosphotransferase EI, Hpr, and EIIA components	2.7.1.69
0496	Sugar transport protein	
1504, 0131, 0084	Possible glucokinase	2.7.1.2
0227	Glucose-6-phosphate dehydrogenase	1.1.1.49
0228	6-Phosphogluconolactonase	2.1.1.31
0232	6-Phosphogluconate dehydrogenase	1.1.1.44
0218	Ribulose-phosphate 3-epimerase	5.1.3.1
1870	Ribose-5-phosphate isomerase A	5.3.1.6
1580	Ribose-5-phosphate isomerase B	5.3.1.6
0265, 170+171	Transketolase	2.2.1.1
0582	Transaldolase	2.2.1.2
1425	G6P isomerase	5.3.1.9
0438	Probable fructose-6-phosphate	4.1.2.-
1439–41 (complete) 1585–6 (M-subunit missing)	Dihydroxyacetone kinase	2.7.1.121
0687	6-Phosphofructokinase	2.7.1.11
0536	Fructose-bisphosphate aldolase	4.1.2.13
0677, 1575, 1601	Triose phosphate isomerase	5.3.1.1
1027	Glyceraldehyde-3-phosphate dehydrogenase	1.2.1.12
0535	Phosphoglycerate kinase	2.7.2.3
0219	Phosphoglycerate phosphatase	
0572	Probable phosphoglycerate mutase	5.4.2.1
1383	Probable phosphoglycerate mutase	
1869	D-3 phosphoglycerate dehydrogenase	
2051	Enolase	4.2.1.11
1171	Pyruvate kinase	2.7.1.40
0005	Putative FAD-binding, membrane bound, D-lactate dehydrogenase	1.1.1.28
0207	Pyruvate formate-lyase	2.3.1.54
1261–6	Formate dehydrogenase	1.2.1.2
0942	Pyruvate dehydrogenase E1	1.2.4.1
0943	Pyruvate dehydrogenase E2	2.3.1.12
0944	Pyruvate dehydrogenase E3; Dihydrolipoamide dehydrogenase	1.8.1.4
0591	Probable acetaldehyde/alcohol dehydrogenase	1.2.1.10, 1.1.1.1
0403, 1136, 1955	Predicted alcohol dehydrogenase	1.1.1.1

0067	Class III Formaldehyde/alcohol dehydrogenase	1.1.1.284, 1.1.1.1
1065	Predicted aldehyde dehydrogenase	1.2.1.3
1662	Phosphotransacetylase	2.3.1.8
1661	Acetate kinase	2.7.2.1
0221	Phosphoenolpyruvate carboxykinase	4.1.1.49
0301-3	Oxaloacetate decarboxylase	4.1.1.3
0669	Putative $NADP^+$-malic enzyme	1.1.1.40
1612	NAD^+-malate dehydrogenase	1.1.1.37
0956	Fumarase class II	4.2.1.2
1813-6	Fumarate reductase	1.3.99.1
1564-5	Putative succinyl-CoA synthetase	6.2.1.5
1567	α-Ketoglutarate dehydrogenase E1	1.2.4.2
1566	α-Ketoglutarate dehydrogenase E2	2.3.1.61
0944	α-Ketoglutarate dehydrogenase E3	1.8.1.4
0185	Aconitase	4.2.1.3
0305, 1194–6, 1198	Citrate lyase	4.1.3.6
1277–83	Putative [Ni-Fe]-hydrogenase	1.12.7.2
1199	Carbonic anhydrase	4.2.1.1
1021–2	Transhydrogenase	1.6.1.1, 1.6.1.2
1073	Putative fructose-1,6-bisphosphatase (Type I)	3.1.3.11
0394	Fructose-1,6-bisphosphatase (Type II)	3.1.3.11

Table S4. Partial biosynthetic pathways present in *A. succinogenes* for required amino acids and vitamins.

Asuc_ #	Function	*A. succinogenes* modified product description[a]	E.C. #
	**Assimilatory sulfate reduction**		
1689	Sulfate adenylyltransferase, small subunit, CysD	Probable	2.7.7.4
1688	Sulfate adenylyltransferase, large subunit, Cys N	Probable	2.7.7.4
None	Adenylylsulfate kinase, Cys C	NA	2.7.1.25
1690	Phosphoadenylyl-sulfate reductase CysH	Probable	1.8.4.8
1691	Siroheme synthase CysG (needed for sulfite reductase)	Probable	
1686	Sulfite reductase (NADPH) hemoprotein, β subunit, CysI	Function	1.8.1.2
1687	Sulfite reductase [NADPH] flavoprotein, α-component, CysJ	Function	1.8.1.2
1692	Periplasmic sulfate/thiosulfate binding protein	Function	
1693	Sulfate ABC transporter, permease protein, CysT	Function	
1694	Sulfate ABC transporter, permease protein CysW	Function	
1695	Sulfate/thiosulfate ABC transporter, ATP-binding protein	Function	
	**Cysteine biosynthesis**		
0384	Serine acetyltransferase, CysE	Putative	2.3.1.30
2108	Cysteine synthase A, CysK	Probable	2.5.1.47
	**Methionine biosynthesis**		
1733	Homoserine O-acetyltransferase	Function	2.3.1.31
None	Homoserine O-succinyltransferase metA	NA	2.3.1.46
1846	O-succinylhomoserine lyase	Possible	2.5.1.48
1623	O-acetylhomoserine aminocarboxypropyltransferase	Function	2.5.1.49
1135	Cystathionine β-lyase	Function	4.4.1.8
None	Cobalamin-dependent homocysteine transmethylase, MetH	NA	2.1.1.13
None	Cobalamin-independent homocysteine transmethylase	NA	2.1.1.14
1415	Homocysteine S-methyltransferase	Function	2.1.1.10
	**Biotin**		
None	6-Carboxyhexanoate--CoA ligase BioW	NA	6.2.1.14
None	8-Amino-7-oxononanoate synthase BioF	NA	2.3.1.47
None	Adenosylmethionine--8-amino-7-oxononanoate transaminase BioA	NA	2.6.1.62
None	Carboxylesterase BioH	NA	3.1.1.1
None	Biotein synthesis protein BioC	NA	
None	Biotin biosynthesis cytochrome P450	NA	1.14.-.-
None	BioM	NA	
None	BioN	NA	

None	BioY	NA	
1117	Dithiobiotin synthetase BioD	Possible	6.3.3.3
1132	Biotin synthase BioB	Probable	2.8.1.6
	**Nicotinic acid**		
None	L-aspartate oxidase NadB	NA	1.4.3.16
None	Quinolinate synthetase complex, A subunit NadA	NA	4.6.1.3
None	Nicotinate-nucleotide diphosphorylase (carboxylating) NadC	NA	2.4.2.19
1925	NAD nucleotidase UshA	Probable	3.1.3.5
0624	Purine-nucleoside phosphorylase DeoD	Function	2.4.2.1
0487	Bifunctional DNA-binding transcriptional repressor/nicotinamide-nucleotide adenylyltransferase NadR	Function	2.7.7.1
0640	NAD^+ synthetase NadE	Possible	6.3.1.5
1021	$NAD(P)^+$ transhydrogenase, alpha subunit PntA	Putative	1.6.1.1
1022	$NAD(P)^+$ transhydrogenase, beta subunit PntB	Function	1.6.1.2
	**Pantothenate**		
1039	acetolactate synthase III, large subunit IlvI	Putative	2.2.1.6
1040	acetolactate synthase III, thiamin-dependent, small subunit IlvH	Putative	2.2.1.6
0563	ketol-acid reductoisomerase, NAD(P)-binding IlvC	Function	1.1.1.86
0415	dihydroxyacid dehydratase IlvD	Function	4.2.1.9
None	3-Methyl-2-oxobutanoate hydroxymethyltransferase PanB	NA	2.1.2.11
None	2-Dehydropantoate 2-reductase PanE	NA	1.1.1.169
None	Pantothenate synthetase, pantoate-β-alanine ligase PanC	NA	6.3.2.1
0095	Pantothenate kinase PanK	Function	2.7.1.33
0012	Phosphopantothenoylcysteine decarboxylase/phosphopantothenate--cysteine ligase CoaBC	Function	4.1.1.36, 6.3.2.5
0675	Pantotheine-phosphate adenylyltransferase CoaD	Function	2.7.7.3
0609	Dephospho-CoA kinase CoaE	Function	2.7.1.24
	**Pyridoxine**		
[b]1027	Glyceraldehyde-3-phosphate dehydrogenase GapA	Function	1.2.1.72
[c]1869	Erythronate-4-phosphate dehydrogenase PdxB	Possible	1.1.1.290
0922	Phosphoserine aminotransferase PdxC	Function	2.6.1.52
1953	4-Hydroxythreonine-4-phosphate dehydrogenase PdxA	Function	1.1.1.262
1372	1-Deoxy-D-xylulose-5-phosphate synthase Dxs	Function	2.2.1.7
None	Pyridoxine 5'-phosphate synthase PdxJ	NA	2.6.99.2
0247	Pyridoxamine 5'-phosphate oxidase PdxH	Function	1.4.3.5
None	Glutamine amidotransferase	NA	2.6.-.-
None	Pyridoxine biosynthesis protein	NA	4.-.-.-

[a] Putative, 60–75% amino acid identity; Probable, 40–59% amino acid identity; Possible, 25–39% amino acid identity; NA, no suitable homolog identified in *A. succinogenes* either due to insufficient length of alignment (less than 25% of the query sequence length) or to no hits retrieved from the BLAST search.

[b] E.C. 1.2.1.72 corresponds to erythrose-4-phosphate dehydrogenase (Epd) activity. In *E. coli*, both GapA and Epd have erythrose-4-phosphate dehydrogenase activity [1]. GapA alone was also shown to be sufficient for pyridoxal 5'-phoshphate biosynthesis in the absence of Epd [2].

[c] Asuc1869 (Putative SerA, D-3-phosphoglycerate dehydrogenase) shares 28 % identity (over 72 % of the protein length) with the *E. coli* PdxB protein. SerA has been suggested to be an alternate enzyme for erythronate-4-phosphate dehydrogenase activity in the pyridoxine biosynthesis pathway [3, 4].

1. BoschiMuller S, Azza S, Pollastro D, Corbier C, Branlant G: **Comparative enzymatic properties of GapB-encoded erythrose-4-phosphate dehydrogenase of *Escherichia coli* and phosphorylating glyceraldehyde-3-phosphate dehydrogenase**. *J Biol Chem* 1997, **272**:15106–15112.

2. Yang Y, Zhao GS, Man TK, Winkler ME: **Involvement of the *gapA*- and *epd* (*gapB*)-encoded dehydrogenases in pyridoxal 5 '-phosphate coenzyme biosynthesis in *Escherichia coli* K-12**. *J Bacteriol* 1998, **180**:4294–4299.

3. Arps PJ, Winkler ME: **An unusual genetic link between vitamin B6 biosynthesis and tRNA pseudouridine modification in *Escherichia coli* K-12**. *J Bacteriol* 1987, **169**:1071–1079.

4. Lam HM, Winkler ME: **Metabolic relationships between pyridoxine (vitamin B6) and serine biosynthesis in *Escherichia coli* K-12**. *J Bacteriol* 1990, **172**:6518–6528.

Table S5. *A. succinogenes* dicarboxylate transporters. TRAP: tripartite ATP-independent periplasmic.

**Asuc ORF #**	**Dicarboxylate transporter**
1577	Possible TRAP C4-dicarboxylate transporter, large permease component, DctM (31%)
1578	Conserved hypothetical protein (TRAP, small permease component, DctQ) (<25%)
1579	Possible TRAP C4-dicarboxylate transporter solute receptor, DctP (26%)
0148	Possible TRAP C4-dicarboxylate transporter, large permease component, DctM (34%)
0147	Possible TRAP C4-dicarboxylate transporter, small permease component, DctQ (35%)
0146	Possible TRAP C4-dicarboxylate transporter solute receptor, DctP (30%)
0156	Possible TRAP C4-dicarboxylate transporter, large permease component, DctM (34%)
0157	Conserved hypothetical protein (TRAP, small permease component, DctQ) (<25%)
0158	Possible TRAP C4-dicarboxylate transporter solute receptor, DctP (26%)
0368	Possible TRAP C4-dicarboxylate transporter, large permease component, DctM (31%)
0367	Conserved hypothetical protein (TRAP, small permease component, DctQ)
0366	Possible TRAP C4-dicarboxylate transporter solute receptor, DctP (28%)
1921	Possible TRAP C4-dicarboxylate transporter, large permease component, DctM (35%)
1922	Possible TRAP C4-dicarboxylate transporter, small permease component, DctQ (31%)
1923	Possible TRAP C4-dicarboxylate transporter solute receptor, DctP (28%)
1165	Possible TRAP C4-dicarboxylate transporter, large permease component, DctM (33%)
1164	Possible TRAP C4-dicarboxylate transporter, small permease component, DctQ (35%)
1163	Possible TRAP C4-dicarboxylate transporter solute receptor, DctP (29%)
1957	TRAP transporter, 4TM/12TM fusion protein (based on TIGRFAM subfamily)
1958	TRAP transporter solute receptor, TAXI family (based on TIGRFAM subfamily)
1988	TRAP transporter solute receptor, TAXI family (based on TIGRFAM subfamily)
1990	Possible TRAP C4-dicarboxylate transporter, large permease component, DctM (4TM/12TM fusion protein) (34%)
1991	TRAP transporter solute receptor, TAXI family (based on TIGRFAM subfamily)
0273	Possible TRAP C4-dicarboxylate transporter solute receptor, DctP (31%)
0272	Possible TRAP C4-dicarboxylate transporter solute receptor, DctP (29%)
0271	Possible TRAP C4-dicarboxylate transporter, large permease component, DctM
0270	Conserved hypothetical protein (TRAP, small permease component, DctQ) (21%)
0142	Probable anaerobic C4-dicarboxylate transporter DcuB or DcuA (T.C.2.A.1.3) (41% ident to *E. coli* DcuA, 42% to DcuB)<br>TIGRFAM: DcuA is usually found in an operon with aspartase and DcuB with fumarase; Asuc _0142 is next to the aspartase gene (Asuc_0141) but the two genes are in opposite directions
1999	Putative anaerobic C4-dicarboxylate transporter DcuB (74%)
1063	Possible anaerobic C4-dicarboxylate transporter DcuC (T.C. 2.A.6.1)
