# Supplementary material for: A genomic perspective on the potential of Actinobacillus succinogenes for industrial succinate production
Source: BMC Genomics. 2010 Nov 30;11:680. doi: 10.1186/1471-2164-11-680 (PMC3091790; doi:10.1186/1471-2164-11-680)
Supplement: Additional file 4 — Explanation for lack of pathogenicity--extended discussion. The supplementary text contains an extended discussion of alignment of Pasteurellaceae virulence factors against succinogens' genomes. The discussion touches on many of the negative results not reported in the main text. [file 1471-2164-11-680-S4.PDF]

Supplementary text.

Explanation for lack of pathogenicity – extended discussion.
The natural Pasteurellaceae ecology is in association with a host [1]. The host is usually mammalian, with a few exceptions [2], such as *P. multocida* colonizing birds [1] and possibly even amoebas [3]. Most Pasteurellaceae can be isolated from healthy hosts and are considered part of the normal flora. However, in circumstances such as host stress, many Pasteurellaceae cause disease and are considered opportunistic pathogens. While the American Type Culture Collection lists *A. succinogenes* as a biosafety level 1 organism, all members of the genus *Actinobacillus* are automatically classified as risk group 2 bacterial agents by the National Institute of Health, and require handling under biosafety level 2 guidelines [4]. Most Pasteurellaceae are isolated from the respiratory tract and cause pulmonary diseases [1]. Others have been isolated from the oral cavity (e.g., *A. actinomycetemcomitans*, which causes peridontitis), the genital tract (e.g., *H. ducreyi*, which causes sexually-transmitted chancroid), and bovine rumen (e.g., *A. lignieresii*, which causes wooden tongue) [2]. Virulence is an undesirable trait for an industrial organism, and the relatedness of *A. succinogenes* and *M. succiniciproducens* to several pathogens cannot be ignored. With no reports of disease caused by the succinogens, their genome sequences are a convenient and logical starting point to assess their potential for lack of pathogenicity.

Many virulence factors have been characterized in Pasteurellaceae species. We manually compiled a list of Pasteurellaceae virulence proteins and aligned them against the *A. succinogenes* and *M. succiniciproducens* protein databases (supplementary Table 6). Genome-level approaches, such as microarrays [5-7] and signature-tagged transposon mutagenesis [8, 9], indicated that Pasteurellaceae virulence is associated with global metabolic changes, including expression of amino acid transporters, purine and pyrimidine biosynthesis enzymes, and enzymes for anaerobic metabolism. While these three groups of metabolic activities may affect host health, they are also necessary for nonvirulent processes. Genes in these categories were excluded from the comparison. Our list includes 259 gene products (with some redundancy) with functions in toxin production, synthesis of cell surface structures, and iron uptake (supplementary Table 6). A similar list comparing Pasteurellaceae virulence factors was recently reported by Challacombe and Inzana [10]; it partially overlaps ours. We also aligned the non-overlaping members of the Challacombe and Inzana list against the succinogens' genomes (total of 341 gene products for the combined lists). In some cases our findings disagree with those of Challacombe and Inzana. All disagreements, though, are for protein query sequences that aligned over less than 25% of the length of the top succinogen hit, and thus did not meet our criteria for having the same function. These differences could be explained by Challacombe and Inzana using query sequences and

selection criteria different from ours. The major findings from the combined lists are summarized in this section with more details available in supplementary Table 6.

Toxins. Repeat toxins (RTX), including leukotoxins, are produced by many Pasteurellaceae, including *A. pleuropneumoniae* [11], *A. actinomycetemcomitans* [12], *M. haemolytica* [13], and *P. multocida* [14]. The rumen bacterium *A. lignieresii* has a full RTX toxin operon, but it lacks a promoter region to express it [14]. Leukotoxin production relies on four proteins: protein A, the structural pretoxin; protein C, the pretoxin activator; and proteins B and D, which form a type I secretion system [14]. The pretoxin is not encoded in either succinogen. Only possible homologs to the B and D proteins are found, likely due to conserved sequences for ATP-dependent transport. It is important to note that the B and D homologs are never found in the same operon in the succinogens, whereas the four leukotoxin genes are normally organized in an operon. Possible pretoxin hits in *M. succiniciproducens* clearly encode proteins of non-pathogenic functions (e.g., shikimate 5-dehydrogenase; YP_087630.1). The absence of the pretoxin strongly suggests that neither succinogen can produce a leukotoxin. A possible hit against the pretoxin-activating protein in *M. succiniciproducens* (YP_088360.1) is encoded in a gene cluster that is not involved in leukotoxin production but may be involved in hemagglutinin production (YP_088354-61.1; see next section). *A. actinomycetemcomitans* uses a cytolethal distending toxin that is encoded near a characteristic virulence-associated region [15]. Neither succinogen genome sequence encodes the cytolethal distending toxin. Neither succinogen encodes a homolog to the *H. ducreyi* hemolysin [16].

Cell surface structures. Cell surface virulence factors used by pathogenic Pasteurellaceae include pili, adhesins, LPS, and capsules. Adherence to respiratory epithelial cells is the first colonization stage by respiratory Pasteurellaceae pathogens. Even though the succinogens were isolated from the bovine rumen, their large-scale culturing could pose a respiratory infection risk due to aerosolization. Adherence involves a number of cell surface mechanisms [17]. Both succinogens have possible homologs to OapA and B, which are involved in *H. influenzae* binding to epithelial cells [18]. Neither succinogen encodes *H. influenzae's* surface fibril protein, Hsf [19]. Both succinogens have all components (with varying levels of similarity) of the type IV pilus (*pilABCD*) involved in *H. influenzae's* adherence to nasopharyngeal tissue [20]. These pili are also part of the *H. influenzae* competence regulon [21] and both competence and pathogenicity are regulated in part by stress. *M. succiniciproducens* has probable homologs of the *A. actinomycetemcomitans* pili needed for tight adherence (*flp* and *tad* loci) [22], whereas *A. succinogenes* does not. Neither succinogen encodes homologs of the *H. influenzae* adherence and penetration protein, Hap [17] and of the *A. actinomycetemcomitans* collagen adhesin, EmaA [23]. *H. influenzae* strains use high molecular weight (HMW)

adhesin proteins to colonize respiratory tissues [17, 24, 25]. Most *H. influenzae* HMW adhesins do not have significant sequence identity to any succinogen ORF, aside from putative HmwC homologs in both genomes. Both succinogens have several large ORFs that could encode HMW adhesins, including the possible *M. succiniciproducens* hemagglutinin gene cluster mentioned above. *A. succinogenes* also has a gene cluster that may be involved in hemagglutinin production (Asuc_1006–12), but ORFs Asuc_1006 and 1008 have internal frame shifts. Does *A. succinogenes* make an adhesin? Tests must be done to find out. It is also possible that *A. succinogenes* uses this feature for survival in a competitive environment, rather than for causing disease.

Homologs of *H. influenzae's* hemagglutinating pilus genes (*hifA-E*) are not found in the succinogens, with the exception of a possible chaperonin-like HifB component in both. Although the criteria used by Challacombe and Inzana found that *M. succiniciproducens* has proteins similar to hemagglutinins FhaB, pFHB1, and pFHB2 [10], these proteins did not pass the alignment length requirement we set (at least 25%) to be considered homologs to FhaB, pFHB1 and pFHB2.

Gram-negative bacteria possess an outer membrane LPS that can act as an endotoxin and that can play a role in evading the immune system through choline and sialic acid incorporation. The succinogens' genome sequences contain many genes involved in LPS synthesis and modification (supplementary Table 6). None of these genes contain variable number tandem repeats, suggesting that the succinogens are not capable of LPS phase variation. Nontypeable *H. influenzae* strains are able to evade host immune defenses by incorporating host sialic acid and choline into their LPSs, thereby mimicking host cell surfaces [26, 27]. Both succinogens lack the genes for choline incorporation (*licABCD*) [20], with the exception of a possible *licB* gene in *A. succinogenes*. It is also doubtful that either succinogen is capable of sialic acid incorporation. While they have possible homologs to the tripartite ATP-independent periplasmic-like sialic acid transporter, they do not have the sialic acid-binding protein (SiaP), the regulatory protein (SiaR), or the sialic acid incorporation proteins, Lic3A2 and SiaB [28].

A. succinogenes' LPS could be more complex than that of *M. succiniciproducens*, since *A. succinogenes* has several LPS glycosyltransferases not found in *M. succiniciproducens* (e.g., Asuc_0524, 1375). The two succinogens' LPSs might also differ in their sugar compositions. L-Rhamnose is commonly found in the cell wall and the capsule of many bacteria, and thymidine diphosphate (dTDP)-L-rhamnose is the immediate source of rhamnose in carbohydrate polymers [29]. Four key enzymes, RmlA to D, are required for dTDP-L-rhamnose synthesis from glucose-1-phosphate and thymidine triphosphate [29]. *A. succinogenes*, *A. acetomycetemcomitans*, *A.*

pleuropneumoniae, and *A. minor* are the only Pasteurellaceae species containing homologs of *E. coli* RmlA, B, C, and D. In *A. succinogenes*, Asuc_0826 (product 75% identical to *E. coli* RmlB), Asuc_0829 (product 65% identical to *E. coli* RmlA), Asuc_0830 (product 44% identical to *E. coli* RmlC), and Asuc_0832 (product 53% identical to *E. coli* RmlD) seem to be organized in one operon. In this operon, Asuc_0827 and Asuc_828 are possibly encoding another nucleotidyl transferase of unknown specificity. Asuc_0827 and Asuc_0828 are 51% and 50% identical to *H. influenzae* HI0074 and HI0073, respectively, which are likely the substrate-binding (HI0074) and nucleotide-binding (HI0073) domains of a new family of nucleotidyltransferases [30, 31]. The function of the *A. succinogenes* L-rhamnose biosynthetic pathway could be related to lipopolysaccharide (LPS) synthesis, since Asuc_0826−32 are located just downstream of Asuc_0821−24, which encode enzymes considered to be involved in LPS biosynthesis, and since L-rhamnose is a common component of the LPS O-antigen [32, 33]. Because LPS O antigens are mostly studied in pathogenic bacteria, it is unclear how often non-pathogenic bacteria contain rhamnose in their LPS. For this reason, the possible presence of rhamnose in *A. succinogenes* LPS is by no means indicative of a virulence trait. In contrast to *A. succinogenes*, *M. succiniciproducens* contains only a RmlB homolog (MS1593; 80% identity to Asuc_0826). *M. succiniciproducens* contains instead a set of ORFs encoding proteins likely involved in L-rhamnose transport (RhaT, MS2326) and catabolism (RhaBAD, MS2327−29), as well as the L-rhamnose-dependent regulators RhaS (MS2322) and RhaR (MS2323). None of these genes are found in *A. succinogenes*. Thus, the two succinogens have evolved completely different L-rhamnose pathways—a biosynthetic one in *A. succinogenes* and a catabolic one in *M. succiniciproducens*.

P. multocida, A. pleuropneumoniae, *M. haemolytica*, and typeable *H. influenzae* produce a capsule that is important for virulence [34-38]. Both succinogens have possible homologs to, at most, two of the four capsule biosynthesis and export proteins, suggesting that they are not capsulated bacteria (supplementary Table 6). However, non-typeable *H. influenzae* are non-capsulated but they are still virulent.

Iron uptake mechanisms. While iron acquisition is a well-documented Pasteurellaceae virulence trait, it is a common trait in most bacteria, making it difficult to associate iron uptake with virulence. Nonetheless, some insight can be gained from the form of iron transported. For example, transferrin and hemoglobin are proteins that mammalian hosts use to bind iron. Unlike transferrin- and hemoglobin-utilizing *A. pleuropneumoniae* [34, 39], *H. influenzae* [20], *H. somni* [40], *M. haemolytica* [41], and *P. multocida* [42], the succinogens have possible homologs to only a few of the proteins required for transferrin (TbpAB), heme/hemopexin (HxuABC), or hemoglobin uptake (HgpABC) (supplementary Table 6). Possible hits against the *H. influenzae hitABC* [20] system share much higher sequence identity with polyamine transporters, and are thus not likely true

hitABC homologs. *A. succinogenes* does not have homologs to the hemin receptor HemR or to the heme utilization protein Hup [20], while *M. succiniciproducens* has possible homologs of each. Neither succinogen has a homolog to the *M. haemolytica* hypermutable iron receptor protein Irp [43] or to the *H. influenzae* HfeABCD system, which putatively transports chelated iron [20]. Still, BLAST searches show that both succinogens can assimilate other forms of iron, including iron bound by various siderophores (supplementary Table 6), and that they contain the heme biosynthetic pathway from L-glutamate (Asuc_1116, 1761, 0667, 0356, 0355, 1982, 0382, 1871, 0513, and 1562 in *A. succinogenes* [44] (supplementary Table 6). It is interesting that in both succinogens the potential hemagglutinin production system mentioned above is encoded alongside genes involved in ferrous iron transport, including *feoAB*. FeoA and B are not encoded in any sequenced Pasteurellaceae other than the succinogens and *A. minor* NM305, but they have been implicated in more distantly related bacteria in virulence and colonization of mammalian intestines [45, 46]. This genetic region may be a worthwhile target for deletion, provided it does not contain essential genes for growth and succinate production.

Other virulence proteins. Both succinogens encode GroEL homologs. While GroEL is involved in bone resorption by *A. actinomycetemcomitans* [12], it is also a ubiquitous chaperonin that is important for proper protein folding [47]. Both succinogens have a putative homolog to the inner membrane protein, ImpA, involved in autoaggregation [12]. Neither has a putative homolog to the *A. actinomycetemcomitans* immunosuppressive factor, SF1 [48], or to its Type IV secretion system [49]. Some Pasteurellaceae have urease activity, which is a known virulence factor of gastroduodenal and urinary tract pathogens [50], but the succinogens have no urease homologs, and *A. succinogenes* tested negative for urease activity [51]. There is also no homolog in either succinogen genome sequence to the *H. influenzae* Iga protease, which cleaves immunoglobulin A1, helping *H. influenzae* avoid host defenses at mucosal surfaces [52, 53].

We want to stress that nonpathogenicity cannot be concluded from the analysis of a genome sequence. Most Pasteurellaceae species cause respiratory diseases. The succinogens are less likely to do so, since they live in the rumen. The virulence factors associated with a hypothetical succinogen-caused disease could be different from those used by other Pasteurellaceae. For example, the FeoAB iron uptake system important for the virulence of some intestinal pathogens is unique to the succinogens in the thirty-two partially and fully sequenced Pasteurellaceae, with *A. minor* NM305 (part of the pig respiratory tract normal flora) an exception. This system, though, could also be important for a commensal relationship with the mammalian host.

References

1. Killian M, Frederiksen W, Biberstein EL: ***Haemophilus, Pasteurella,* and *Actinobacillus***. New York: Academic Press Inc.; 1981

2. Christensen H, Bisgaard M: **Taxonomy and biodiversity of members of *Pasteurellaceae***. In: *Pasteurellaceae: Biology, Genomics, and Molecular Aspects.* Edited by Kuhnert P, Christensen H. Norfolk, UK: Caister Academic Press; 2008: 1–26

3. Hundt MJ, Ruffolo CG: **Interaction of *Pasteurella multocida* with free-living amoebae**. *Appl Environ Microbiol* 2005, **71**:5458–5464

4. **NIH Guidelines for research involving recombinant DNA molecules (NIH Guidelines)**: National Institutes of Health; 2002

5. Boyce JD, Wilkie I, Harper M, Paustian ML, Kapur V, Adler B: **Genomic scale analysis of *Pasteurella multocida* gene expression during growth within the natural chicken host**. *Infect Immun* 2002, **70**:6871–6879

6. Boyce JD, Wilkie I, Harper M, Paustian ML, Kapur V, Adler B: **Genomic-scale analysis of *Pasteurella multocida* gene expression during growth within liver tissue of chickens with fowl cholera**. *Microbes Infect* 2004, **6**:290–298

7. Wong SM, Akerley BJ: **Environmental and genetic regulation of the phosphorylcholine epitope of *Haemophilus influenzae* lipooligosaccharide.** *Mol Microbiol* 2005, **55**:724–738

8. Sheehan BJ, Bossé JT, Beddek AJ, Rycroft AN, Kroll JS, Langford PR: **Identification of *Actinobacillus pleuropneumoniae* genes important for survival during infection in its natural host**. *Infect Immun* 2003, **71**:3960–3970

9. Ojha S, Sirois M, Macinnes JI: **Identification of *Actinobacillus suis* genes essential for the colonization of the upper respiratory tract of swine**. *Infect Immun* 2005, **73**:7032–7039

10. Challacombe JF, Inzana TJ: **Comparative genomics of *Pasteurellaceae***. In: *Pasteurellaceae Biology, Genomics, and Molecular Aspects.* Edited by Kuhnert P, Christensen H. Norfolk, UK: Caister Academic Press; 2008: 53–77

11. Schaller A, Kuhn R, Kuhnert P, Nicolet J, Anderson TJ, MacInnes JI, Segers RP, Frey J: **Characterization of *apxIVA,* a new RTX determinant of *Actinobacillus pleuropneumoniae***. *Microbiology* 1999, **145**:2105–2116

12. Henderson B, Nair SP, Ward JM, Wilson M: **Molecular pathogenicity of the oral opportunistic pathogen *Actinobacillus actinomycetemcomitans***. *Annu Rev Microbiol* 2003, **57**:29–55

13. Davies RL, Whittam TS, Selander RK: **Sequence diversity and molecular evolution of the leukotoxin (*lktA*) gene in bovine and ovine strains of *Mannheimia* (*Pasteurella*) *haemolytica***. *J Bacteriol* 2001, **183**:1394–1404

14. Schaller A, Kuhnert P, de la Puente-Redondo VA, Nicolet J, Frey J: **Apx toxins in *Pasteurellaceae* species from animals**. *Vet Microbiol* 2000, **74**:365–376

15. Mayer MP, Bueno LC, Hansen EJ, DiRienzo JM: **Identification of a cytolethal distending toxin gene locus and features of a virulence-associated region in *Actinobacillus actinomycetemcomitans***. *Infect Immun* 1999, **67**:1227–1237

16. Palmer KL, Munson RSJ: **Cloning and characterization of the genes encoding the hemolysin of *Haemophilus ducreyi***. *Mol Microbiol* 1995, **18**:821–830

17. Ecevit IZ, McCrea KW, Pettigrew MM, Sen A, Marrs CF, Gilsdorf JR: **Prevalence of the *hifBC, hmw1A, hmw2A, hmwC,* and *hia* genes in *Haemophilus influenzae* isolates**. *J Clin Microbiol* 2004, **42**:3065–3072

18. Prasadarao NV, Lysenko E, Wass CA, Kim KS, Weiser JN: **Opacity-associated protein A contributes to the binding of *Haemophilus influenzae* to Chang epithelial cells**. *Infect Immun* 1999, **67**:4153–4160

19. St Geme JWr, Cutter D, Barenkamp SJ: **Characterization of the genetic locus encoding *Haemophilus influenzae* type b surface fibrils**. *J Bacteriol* 1996, **178**:6281–6287

20. Harrison A, Dyer DW, Gillaspy A, Ray WC, Mungur R, Carson MB, Zhong H, Gipson J, Gipson M, Johnson LS *et al*: **Genomic sequence of an otitis media isolate of nontypeable *Haemophilus influenzae*: comparative study with *H. influenzae* serotype d, strain KW20**. *J Bacteriol* 2005, **187**:4627–4636

21. Redfield RJ, Cameron AD, Qian Q, Hinds J, Ali TR, Kroll JS, Langford PR: **A novel CRP-dependent regulon controls expression of competence genes in *Haemophilus influenzae***. *J Mol Biol* 2005, **347**:735–747

22. Planet PJ, Kachlany SC, Fine DH, DeSalle R, Figurski DH: **The widespread colonization island of *Actinobacillus actinomycetemcomitans***. *Nat Genet* 2003, **34**:193–198

23. Mintz KP: **Identification of an extracellular matrix protein adhesin, EmaA, which mediates the adhesion of *Actinobacillus actinomycetemcomitans* to collagen**. *Microbiol* 2004, **50**:2677–2688

24. van Schilfgaarde M, van Ulsen P, Eijk P, Brand M, Stam M, Kouame J, van Alphen L, Dankert J: **Characterization of adherence of nontypeable *Haemophilus influenzae* to human epithelial cells**. *Infect Immun* 2000, **68**:4658–4665

25. Barenkamp SJ, St Geme JWr: **Identification of a second family of high-molecular-weight adhesion proteins expressed by non-typable *Haemophilus influenzae***. *Mol Microbiol* 1996, **19**:1215–1223

26. West-Barnette S, Rockel A, Swords WE: **Biofilm growth increases phosphorylcholine content and decreases potency of nontypeable *Haemophilus influenzae* endotoxins**. *Infect Immun* 2006, **74**:1828–1836

27. Allen S, Zaleski A, Johnston JW, Gibson BW, Apicella MA: **Novel sialic acid transporter of *Haemophilus influenzae***. *Infect Immun* 2005, **73**:5291–5300

28. Johnston JW, Zaleski A, Allen S, Mootz JM, Armbruster D, Gibson BW, Apicella MA, Munson RSJ: **Regulation of sialic acid transport and catabolism in *Haemophilus influenzae***. *Mol Microbiol* 2007, **66**:26–39

29. Giraud MF, Naismith JH: **The rhamnose pathway**. *Curr Opin Struct Biol* 2000, **10**:687–696

30. Lehmann C, Lim K, Chalamasetty VR, Krajewski W, Melamud E, Galkin A, Howard A, Kelman Z, Reddy PT, Murzin AG *et al*: **The HI0073/HI0074 protein pair from *Haemophilus influenzae* is a member of a new nucleotidyl transferase family: structure, sequence analyses, and solution studies**. *Proteins* 2003, **50**:249–260

31. Lehmann C, Pullalarevu S, Krajewski W, Willis MA, Andrey Galkin, Howard A, Herzberg O: **Structure of HI0073 from *Haemophilus influenzae*, the nucleotide-binding domain of a two-protein nucleotidyl transferase**. *Proteins* 2005, **60**:807–811

32. Perry MB, MacLean LL, Gmur R, Wilson ME: **Characterization of the O-polysaccharide structure of lipopolysaccharide from *Actinobacillus actinomycetemcomitans* serotype b**. *Infect Imm* 1996, **64**:1215–1219

33. Perepelov AV, Li D, Liu B, Senchenkova SN, Guo D, Shevelev SD, Shashkov AS, Guo X, Feng L, Knirel YA *et al*: **Structural and genetic characterization of *Escherichia coli* O99 antigen**. *FEMS Immun Med Microbiol* 2009, **57**:80–87

34. Jacques M: **Surface polysaccharides and iron-uptake systems of *Actinobacillus pleuropneumoniae***. *Can J Vet Res* 2004, **68**:81–85

35. Kroll JS, Loynds B, Brophy LN, Moxon ER: **The bex locus in encapsulated *Haemophilus influenzae*: a chromosomal region involved in capsule polysaccharide export**. *Mol Microbiol* 1990, **4**:1853–1862

36. Boyce JD, Adler B: **The capsule is a virulence determinant in the pathogenesis of *Pasteurella multocida* M1404 (B:2)**. *Infect Immun* 2000, **68**:3463–3468

37. Satola SW, Schirmer PL, Farley MM: **Complete sequence of the cap locus of *Haemophilus influenzae* serotype b and nonencapsulated b capsule-negative variants**. *Infect Immun* 2003, **71**:3639–3644

38. Ward CK, Lawrence ML, Veit HP, Inzana TJ: **Cloning and mutagenesis of a serotype-specific DNA region involved in encapsulation and virulence of *Actinobacillus pleuropneumoniae* serotype 5a: concomitant expression of**

serotype 5a and 1 capsular polysaccharides in recombinant *A. pleuropneumoniae* serotype 1. *Infect Immun* 1998, **66**:3326–3336

39. Baltes N, Hennig-Pauka I, Gerlach GF: **Both transferrin binding proteins are virulence factors in *Actinobacillus pleuropneumoniae* serotype 7 infection**. *FEMS Microbiol Lett* 2002, **209**:283–287

40. Challacombe JF, Duncan AJ, Brettin TS, Bruce D, Chertkov O, Detter JC, Han CS, Misra M, Richardson P, Tapia R *et al*: **Complete genome sequence of *Haemophilus somnus* (*Histophilus somni*) strain 129Pt and comparison to *Haemophilus ducreyi* 35000HP and *Haemophilus influenzae* Rd**. *J Bacteriol* 2007, **189**:1890–1898

41. Ogunnariwo JA, Woo TK, Lo RY, Gonzalez GC, Schryvers AB: **Characterization of the *Pasteurella haemolytica* transferrin receptor genes and the recombinant receptor proteins**. *Microb Pathog* 1997, **23**:273–284

42. Bosch M, Garrido ME, Llagostera M, Pérez De Rozas AM, Badiola I, Barbé J: **Characterization of the *Pasteurella multocida hgbA* gene encoding a hemoglobin-binding protein**. *Infect Immun* 2002, **70**:5955–5964

43. Graham MR, Lo RY: **A putative iron-regulated TonB-dependent receptor of *Mannheimia* (*Pasteurella*) *haemolytica* A1: possible mechanism for phase variation**. *Vet Microbiol* 2002, **84**:53–67

44. Panek H, O'Brian MR: **A whole genome view of prokaryotic haem biosynthesis**. *Microbiology* 2002, **148**:2273–2282

45. Naikare H, Palyada K, Panciera R, Marlow D, Stintzi A: **Major role for FeoB in *Campylobacter jejuni* ferrous iron acquisition, gut colonization, and intracellular survival**. *Infect Immun* 2006, **74**:5433–5444

46. Velayudhan J, Hughes NJ, McColm AA, Bagshaw J, Clayton CL, Andrews SC, Kelly DJ: **Iron acquisition and virulence in *Helicobacter pylori*: a major role for FeoB, a high-affinity ferrous iron transporter**. *Mol Microbiol* 2000, **37**:274–286

47. Chapman E, Farr GW, Usaite R, Furtak K, Fenton WA, Chaudhuri TK, Hondorp ER, Matthews RG, Wolf SG, Yates JR *et al*: **Global aggregation of newly translated proteins in an *Escherichia coli* strain deficient of the chaperonin GroEL**. *Proc Natl Acad Sci USA* 2006, **103**:15800–15805

48. Kurita-Ochiai T, Ochiai K: **Immunosuppressive factor from *Actinobacillus actinomycetemcomitans* down regulates cytokine production**. *Infect Immun* 1996, **64**:50–54

49. Novak KF, Dougherty B, Peláez M: ***Actinobacillus actinomycetemcomitans* harbours type IV secretion system genes on a plasmid and in the chromosome**. *Microbiology* 2001, **147**:3027–3035

50. Bossé JT, MacInnes JI: **Genetic and biochemical analyses of *Actinobacillus pleuropneumoniae* urease**. *Infect Immun* 1997, **65**:4389–4394

51. Guettler MV, Rumler D, Jain MK: ***Actinobacillus succinogenes* sp. nov., a novel succinic-acid-producing strain from the bovine rumen**. *Int J Syst Bacteriol* 1999, **49**:207–216

52. Plaut AG: **The IgA1 proteases of pathogenic bacteria**. *Annu Rev Microbiol* 1983, **37**:603–622

53. Poulsen K, Reinholdt J, Kilian M: **A comparative genetic study of serologically distinct *Haemophilus influenzae* type 1 immunoglobulin A1 proteases** *J Bacteriol* 1992, **174**:2913–2921
